# Chemoselective Aza-Michael Addition of Enolizable Heterocyclic Imine-Thiols to Levoglucosenone

**DOI:** 10.3390/molecules31010164

**Published:** 2026-01-01

**Authors:** Anastasia Mauger, Rubi Mahato, Zbigniew J. Witczak, Roman Bielski, Donald E. Mencer

**Affiliations:** 1 Department of Pharmaceutical Sciences, Nesbitt School of Pharmacy, Wilkes University, 84 W. South Street, Wilkes-Barre, PA 18766, USA; anastasia.mauger@wilkes.edu (A.M.); rubi.mahato@wilkes.edu (R.M.); bielski1@verizon.net (R.B.); 2 Department of Chemistry & Biochemistry, Wilkes University, 84 W. South Street, Wilkes-Barre, PA 18766, USA; donald.mencer@wilkes.edu

**Keywords:** levoglucosenone, aza-Michael addition, enolizable imine-thiols, glycorandomization

## Abstract

Heterocyclic sulfur and nitrogen containing compounds capable of forming an equilibrium: thiol/imine = thione/amine (N=C-S-H ⇌ H-N-C=S) were reacted with levoglucosenone (LG) in the presence of triethylamine. Unexpectedly, the only isolated products were the result of the aza-Michael addition. No *S*-adducts were detected. All products were crystalline with good to excellent yields. The structure of products was determined using NMR, MS, and single-crystal X-ray analysis.

## 1. Introduction

Thiols bonded to the five membered rings of thiazole, thiazoline, or thiadiazole are nucleophiles capable of reacting with a conjugated unsaturated system of levoglucosenone **1** [1]. Michael addition could proceed through either the thiol or amine groups to produce the corresponding S or N adducts (or both).

**Scheme 1:**
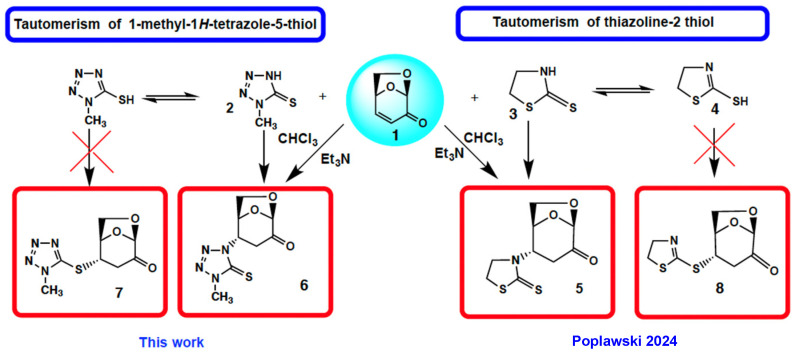
Michael *N*-aza-addition of enolizable heterocyclic thiols to levoglucosenone (LG). The synthesis of compound **5** has been previously described [5].

We have investigated the conjugate Michael addition reaction of thiols for many years [2,3,4], but recently [5] we noticed that heterocyclic iminethiols (N=C-S-H ⇌ H-N-C=S) form only *N*-adducts when reacted in chloroform with levoglucosenone, as depicted in Scheme 1. This is unexpected, since thiols are excellent nucleophiles in Michael additions [6]. Moreover, under certain conditions when thiophenol and aniline addition to acrylonitrile was promoted by triethylammonium acetate, the only product was an *S*-adduct [7].

**Figure 1:**
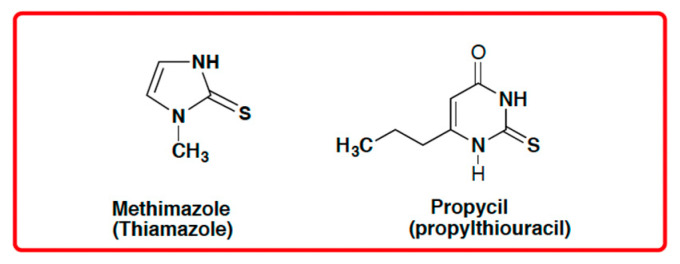
FDA-approved drugs with enolizable thiol functional motifs.

An observation of only *N*-addition of a similar compound (2-mercapto-benzoxazole) to levoglucosenone was previously reported by Spanevello et al. [8]. Recently, 2-mercapto-benzoxazole and 2-mercapto-benzothiazole were used in a Michael addition reaction to α,β-unsaturated esters [9]. Various aspects of thiono-thiol tautomerism of thiadiazole and oxadiazole have also been reported [10,11]. These data and our earlier observation of tautomeric nucleophile 4-thioxo-pyridine acylation [12] prompted us to further investigate the reactivity of enolizable/tautomeric imino-thiols. Interestingly, some substituted thioureas belonging to enolizable/tautomeric imino-thiols are well known FDA-approved drugs, such methimazole (Thiamazole) or propylthiouracil (Propycil), both used to treat hyperthyroidism (Figure 1). Drug disposition, delivery, and toxicity are known to be influenced by the presence of tautomers in some drugs and also by their respective metabolites [13,14].

**Scheme 2:**
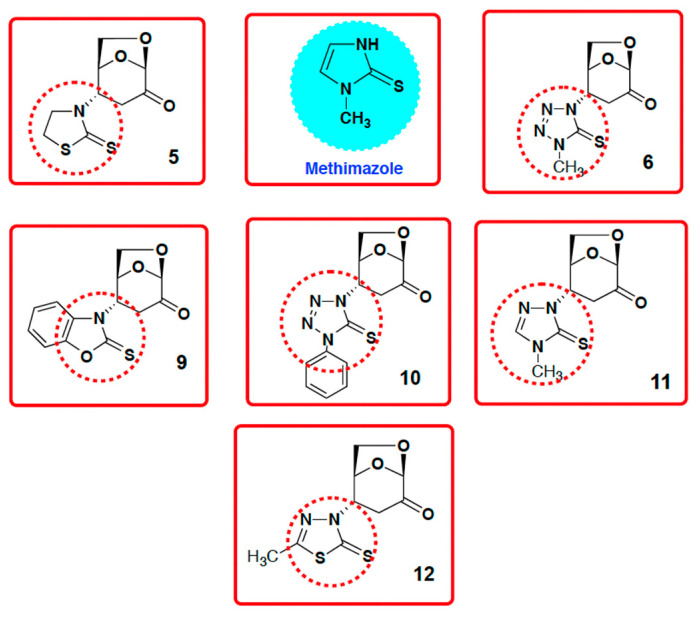
Products of glycorandomization of levoglucosenone after reaction with representative thiols with six different functional motifs (thiazoline, benzoxazole, tetrazole, triazole, and thiadiazole). Note the similarity of methimazole structure to discussed adducts.

The aza-Michael addition of imino-thiols to levoglucosenone (LG) can be considered as a type of glycorandomization [15] in which the LG molecule serves as a representative 1,6-anhydro protected sugar. Glycorandomization [15,16,17] has been reported as a universal platform methodology to modify the physico-chemical character of many natural products, and drugs. Scheme 2 provides an example of glycorandomization of levoglucosenone and representative thiones with six various functional motifs.

Other authors [18] synthesized relevant *N*-adducts via Michael reaction addition to levoglucosenone under aqueous conditions. Interestingly, several of the synthesized levoglucosenone *N*-adducts exhibited promising properties against disorders such as cancer, autoimmune, and heart diseases. Greatrex et al. [19] published some new data on the synthesis of aza LG adducts from the reaction with reactive amines. Mroczek et al. [20] reported some chromatographic characteristics of tautomers of 1,2,4-triazole-3-thione and 3-thiol tautomers. Mloston et al. [21] recently published important studies of the reactivity of enolizable 1-substituted 5-mercapto-1*H*-tetrazoles in the cycloaddition reaction with dimethyl 2-arylcyclopropane dicarboxylates. Ring-opening reactions of donor–acceptor cyclopropanes with enolizable aza-heterocyclic thiones were also reported [22]. Published studies on 1H-benzo[d]imidazole-2-thiols [23] and 2-thioxo-4-thiazolidinone [24] present interesting applications and discuss their structural features and reactivities.

## 2. Results and Discussion

In order to study the addition of enolizable/tautomeric imine-thiols to levoglucosenone (LG), we initiated a study with a series of five-member heterocyclic thiols bearing a nitrogen atom in the α-position to the C=S group, as shown in Table 1. In each case, only *N*-adducts, and no trace of *S*-adducts, were observed as products. Additionally, the stereoselectivity of the addition reaction was influenced by the structure of the levoglucosenone moiety, directing the addition to take place from the bottom of the molecule via *exo*-attack.

**Figure 2:**
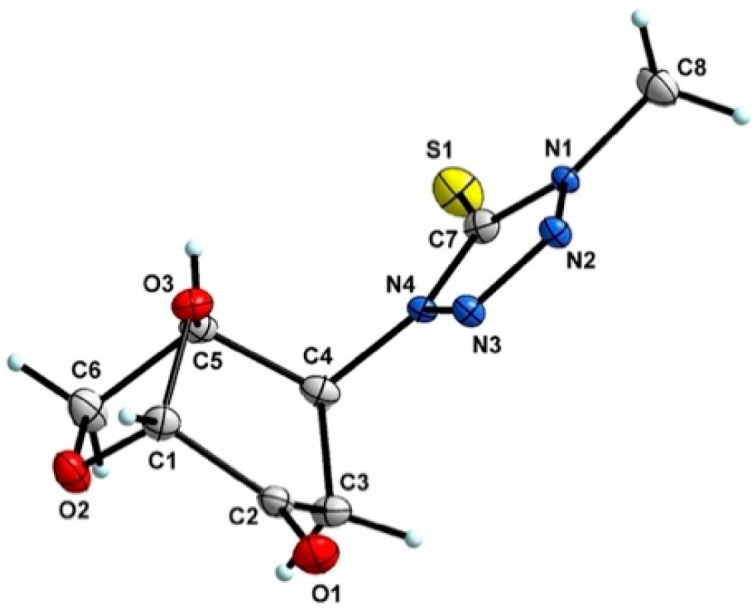
ORTEP diagram of *N*-methyl-2-thioxo-tetrazole (**6**) determined by means of a single-crystal X-ray diffraction. Atoms are represented by thermal ellipsoids (50%) for clarity.

Products were characterized by ^1^H, ^13^C NMR along with single-crystal X-ray diffraction of compound **6**. Crystals of **6** were grown from methanol, and the X-ray analysis revealed the structure presented in Figure 2. The ORTEP diagram for **6** confirms the *N*-linkage of tetrazole moiety connected to C4 of the bicyclic 1,6-anhydrosugar unit, the presence of the thiono group C=S, and the stereospecificity of the addition reaction.

**Scheme 3:**
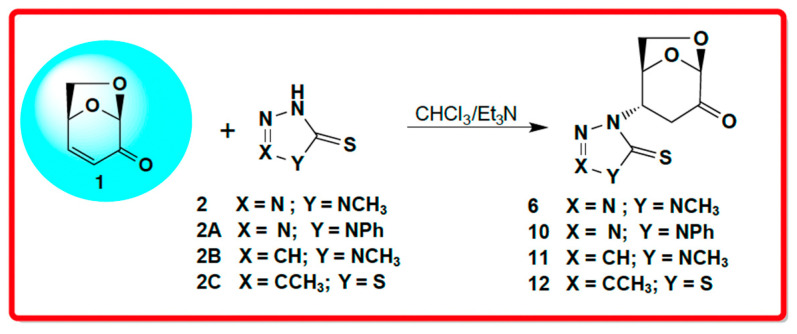
Summary of the aza-Michael addition products with five-membered heterocycles.

The summary of the aza-Michael addition is presented in Scheme 3. One possible explanation for the aza-Michael addition being favored over the thia-Michael addition would be that the starting material exists essentially as only the thione-containing tautomer.

To explore this possibility, we examined the NMR, Raman, and IR spectra of the reacting substrates and found no evidence for the presence of the thiol-enamine tautomer. While it is not definitive proof, this explanation is likely to be correct.

The ^13^C chemical shifts for the thiocarbonyl groups for compounds (**6**, **10**–**12**) were compared with those observed for the enolizable thiols used in this study and chemical shift correlation tables as summarized in Table 2. These specific observations, together with all analytical data, are consistent with the aza-Michael addition reaction of the enolizable heterocyclic thiols to levoglucosenone.

**Scheme 4:**
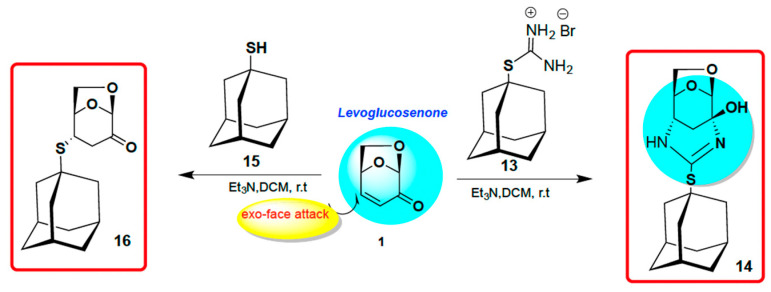
*S*-Adamantane derivative **16**, and *N*-aza adducts of LG **14**, from thiol **15** and masked thiol **13**. Ref. [25]. Published in 2021.

It is worth adding that we also investigated the Michael addition reaction of a masked thiol in the form of thiouronium salt **13** to levoglucosenone [25]. The only product formed under mild basic conditions was polycyclic compound **14**. It must have derived from the initial Michael addition at C4 of LG followed by cyclization to form the six-membered ring. Interestingly, the reaction with corresponding thiol **15** gave the expected Michael ***S***-adduct **16**, as depicted in Scheme 4 [25].

## 3. Material and Methods

CCDC Deposition: Complete crystallographic data for compound **6** have been deposited at the Cambridge Crystallographic Data Centre as supplementary publication numbers CCDC-2212586. These data can be obtained free of charge from the Cambridge Crystallographic Data Centre via https://www.ccdc.cam.ac.uk/structures/ (accessed on 10 December 2025) see Supplementary Materials.

**General information:** All reagents and solvents were used as purchased without further purification.

^1^H NMR and ^13^C NMR spectra were recorded on a Bruker Avance III 400 Ultrashield Plus spectrometer (Bruker BioSpin GmbH, Rheinstetten, Germany). Two-dimensional (COSY and HSQC) experiments were performed to enhance assignments. Chemical shifts (δ–scale) are reported in ppm with TMS (0 ppm) and the residual solvent signals (CDCl_3_: 7.26 ppm) for ^1^H NMR and (CDCl_3_: 77.16 ppm) for ^13^C NMR as internal standards.

Thin-layer chromatography was performed on silica gel-coated TLC plates and visualized under UV light (at 254 nm); detection was executed by exposing to iodine (I_2_) vapor. The melting points (mps) were obtained on an ElectroThermal FARGO MP-2D (Electrothermal Engineering Ltd, Rochford, England, UK) capillary melting point apparatus and were uncorrected. Chemical names were generated by ChemDraw Professional V.15.1.0.144 software.

**Starting materials:** Levoglucosenone (**1**) was prepared from cellulose by sulfuric acid-assisted pyrolysis, following a known procedure [26] and provided as a generous gift from Circa Ltd. (Parkville, Victoria, Australia). All imine-thiols used in the aza-Michael reaction addition were purchased from Sigma-Aldrich (St. Louis, MO, USA).

## 4. Synthetic Procedures and Characterization Data

### General Procedure for the Synthesis of 4-N-Functionalized Dihydrolevoglucosenones

To a solution of levoglucosenone (**1**) 1.26 g (0.01 mole) in 35 mL of chloroform, 0.01 mole of thiol **2** (**2A**–**2C**) was added and magnetically stirred for 5 min. After that time, 1 mL of triethylamine was added dropwise, and the solution was stirred for 24–48 h at room temperature. Upon overnight cooling at 5 °C the crystalline residue was filtered off and recrystallized from methanol. The crystalline products were air-dried.

**Characterization data**

(1*S*,2*S*,5*R*)-2-(4-methyl-5-thioxo-4,5-dihydro-1*H*-tetrazol-1-yl)-6,8-dioxabicyclo[3.2.1]octan-4-one (**6**)


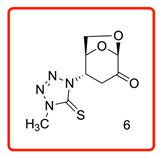


The reaction was carried at room temperature for 24 h. White crystals m. p. 192–194 °C; (1.58 g, 65% yield); R_f_ = 0.26 (Hex/EA 1/1), [α]^D^_30_-54.6 (c 1.0, CHCl_3_); **^1^H NMR** (400 MHz CDCl_3_) δ 2.90 (ddd, *J* = 16.0 Hz, *J* = 5.4 Hz, *J* = 1.4 Hz 1H), 3.19 (dd, *J* = 16.0 Hz, 8.4 Hz, 1H), 3.93 (s, 3H), 4.12 (dd, *J* = 8.4 Hz, *J* = 5.4 Hz, 1H), 4.23 (dd, *J* = 8.4 Hz, *J* = 5.4 Hz, 1 H), 5.05 (d, *J* = 4.0 Hz, 1H), 5.25 (d, *J* = 1.4 Hz, 1H), 5.26 (d, *J* = 2.8 Hz, 1H); **^13^C{1H} NMR** (100 MHz, CDCl_3_) δ 34.67, 34.70, 58.08, 65.88, 73.11, 101.57, 163.59 (C=S), 194.94 (C=O); **APCI-MS** calculated monoisotopic mass for [M+H]+ 243.06 u with detected mass 243.1 u.

(1*S*,2*S*,5*R*)-2-(4-phenyl-5-thioxo-4,5-dihydro-1*H*-tetrazol-1-yl)-6,8-dioxabicyclo[3.2.1]octan-4-one (**10**)


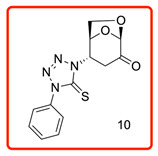


The reaction was carried out under ambient temperature for 48 h. White crystals m. p. 158–160 °C; (1.95 g, 64% yield); R_f_ = 0.22 (Hex/EA 1/1), [α]^D^_30_-51.6 (c 1.0, CHCl_3_); **^1^H NMR** 400 MHz CDCl_3_) δ 2.96 (d, *J* = 17.2 Hz, 1H), 3.24(dd, *J* = 17.2 Hz, 7.6 Hz, 1H), 4.14 (dd *J* = 8.0 *J*z, *J* = 6.0 Hz, 1H), 4.25 (d, *J* = 8.0 Hz, 1H), 5.14 (d, *J* = 4.8 Hz, 1H), 5.32 (s, 1H), 5.39 (d, *J* = 7.6 Hz, 1H), 7.46–7.59 (m, aromatic, 3 H), 7.95 (d, *J* = 8.0 Hz, aromatic 1H); **^13^C{1H} NMR** (100 MHz, CDCl_3_) δ 34.82, 58.00, 65.94, 73.12, 101.63, 123.73, 129.36, 129.88 134.41 162.52 (C=S), 194.95 (C=O); **APCI-MS** calculated monoisotopic mass for [M+H]+ 305.07 u with detected mass 305.1 u.

(1*S*,5*R*)-2-(4-methyl-5-thioxo-4,5-dihydro-1*H*-1,2,4-triazol-1-yl)-6,8-dioxabicyclo[3.2.1]oct-2-en-4-one (**11**)


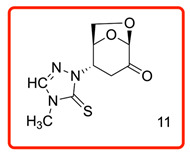


The reaction was carried out under ambient temperature for 24 h. White crystals m. p. 178–180 °C; (1.63 g, 68% yield); R_f_ = 0.29 (Hex/Acetone 1/1), [α]^D^_30_-52.6 (c 1.0, CHCl_3_); **^1^H NMR** (400 MHz CDCl_3_) δ 2.79 (d, *J* = 16.0 Hz, 1H), 3.09 (dd, *J* = 16.0, 7.6 Hz, 1H), 3.62 (s, 3H), 4.07 (dd, *J* = 8.0, *J* = 5.6 Hz, 1H), 4.1 9 (d, *J* = 8.4, Hz, 1H), 5.07 (d, *J* = 4.8 Hz, 1H), 5.23 (s, 1H), 5.34 (d, *J* = 8.0 Hz, 1H), 7.82 (s, 1H). **^13^C{1H} NMR** (100 MHz, CDCl_3_) δ 32.61, 35.01, 58.06, 66.00, 74.07, 101.51, 139.67, 166.15 (C=S), 196.75 (C=O); **APCI-MS** calculated monoisotopic mass for [M+H]+ 242.06 u with detected mass 242.0 u.

(1*S*,2*S*,5*R*)-2-(5-methyl-2-thioxo-1,3,4-thiadiazol-3(2*H*)-yl)-6,8-dioxabicyclo[3.2.1]octan-4-one (**12**)


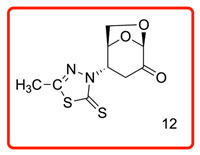


The reaction was carried out at room temperature for 24 h. White crystals m. p. 214–216 °C; (1.85 g, 72% yield); R_f_ = 0.28 (Hex/EA 1/1)), [α]^D^_30_-50.6 (c 1.0, CHCl_3_); **^1^H NMR** (400 MHz CDCl_3_) δ 2.50 (s,3H), 2.81 (dd, *J* = 16.0, 6.4 Hz, 1H), 3.99 (dd, *J* = 15.0, 14.0 Hz), 3.88 (dt, *J* = 12.4, 6.0 Hz, 1H), 4.56 (d, *J* = 8.0 Hz, 1H), 4.94 (br s,1H), 5.20 (s, 1H), 5.77 (dd, *J* =5.2, 4.0 Hz,1H); **^13^C{1H} NMR** (100 MHz, CDCl_3_) δ 34.81, 57.99, 65.93, 73.11, 73.4, 101.62, 123.73, 129.36, 129.87 134.41 162.52, 194.9, 5 (C=O); Minor peaks present are due to an unidentified impurity. **APCI-MS** calculated monoisotopic mass for [M+H]+ 259.01 with detected positive ion [M+H]+ 259.0 u.

## 5. Conclusions

The present study shows that the enolizable/tautomeric imine-thiols **2** (**2A**–**2C**) undergo Michael addition to levoglucosenone (LG) with the formation of *N*-adducts in good yield (64–72%). Only *N*-adducts were isolated and no *S*-adducts were found. In studied solvents the substrates exist only in the amine/thione tautomeric form and, arguably, this is why only the aza-Michael addition products are formed. However, it should be noted that only five-membered heterocyclic thiols were investigated and reported here. Future studies are underway which will attempt to determine under what conditions thia-additions might become favored and why aza additions are dominant in the discussed processes. The results will be published elsewhere.

## Figures and Tables

**Table 1:** Michael addition of enolizable heterocyclic thiols to levoglucosenone.

Substrates	Substrate ^13^C, C=S δ (ppm)	Products	Product ^13^C, C=S δ (ppm)	Product ^13^C, C=O δ (ppm)	m.p. (°C)	Ref.
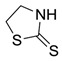	199.21	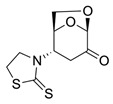 **5**	198.35	198.28	127–129	[2]
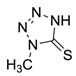 **2**	164.64	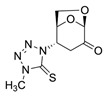 **6**	163.59	194.94	192–194	This work
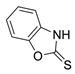	180.58	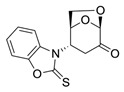 **9**	180.5	197.2	141–142	[5]
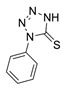 **2A**	164.36	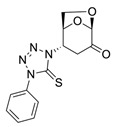 **10**	162.52	194.95	158–160	This work
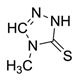 **2B**	166.79	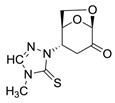 **11**	166.15	196.75	178–180	This work
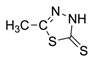 **2C**	189.06	** 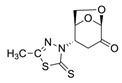 ** **12**	187.30	196.52	214–216	This work

**Table 2:** The ^13^C NMR chemical shifts in thiocarbonyl functional groups as a diagnostic tool.

** 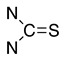 **	** 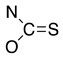 **	** 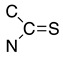 **	** 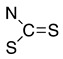 **	** 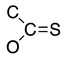 **	** 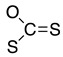 **
δ 160–170	δ 180	δ 190	δ 190–200	δ 195	δ 200–220

## Data Availability

The original contributions presented in this study are included in the article/Supplementary Materials. Further inquiries can be directed to the corresponding author.

## Supplementary Materials

The following supporting information can be downloaded at https://www.mdpi.com/article/10.3390/molecules31010164/s1, X-Ray single crystal analysis and ORTEP of compound **6** and ^1^H, ^13^C NMR spectra.
