# Supplementary material for: Chemoselective Aza-Michael Addition of Enolizable Heterocyclic Imine-Thiols to Levoglucosenone
Source: Molecules. 2026 Jan 1;31(1):164. doi: 10.3390/molecules31010164 (PMC12787532; doi:10.3390/molecules31010164)

Supporting Information for

Chemoselective Aza-Michael addition of enolizable heterocyclic imine-thiols to levoglucosenone.

Anastasia Mauger[a], Rubi Mahato[a] Zbigniew J. Witczak[a]* Roman Bielski[a], Donald E. Mencer[b]

[a]*Department of Pharmaceutical Sciences, Nesbitt School of Pharmacy, and* [b]*Department of Chemistry & Biochemistry, Wilkes University, 84 W. South Street, Wilkes-Barre, PA 18766 Email:* *zbigniew.witczak@wilkes.edu*.

Contents

1. X-Ray single crystal analysis and ORTEP of compound **6**
2. ^{1}H, ^{13}C NMR spectra

Crystal Structure Report for compound 6

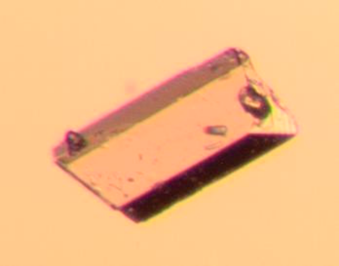

A specimen of $C_8H_{10}N_4O_3S$ was used for the X-ray crystallographic analysis. The X-ray intensity data were measured (λ = 0.71073 Å).

The integration of the data using a monoclinic unit cell yielded a total of 16113 reflections to a maximum θ angle of 30.52° (0.70 Å resolution), of which 3138 were independent (average redundancy 5.135, completeness = 99.9%, R_{int} = 1.53%, R_{sig} = 1.06%) and 3100 (98.79%) were greater than $2\sigma(F^2)$. The final cell constants of a = 17.019(2) Å, b = 6.7825(9) Å, c = 9.8582(13) Å, β = 114.874(2)°, volume = 1032.4(2) $Å^3$, are based upon the refinement of the XYZ-centroids of reflections above 20 σ(I). The calculated minimum and maximum transmission coefficients (based on crystal size) are 0.7156 and 0.7461.

The final anisotropic full-matrix least-squares refinement on F^2 with 185 variables converged at R1 = 2.00%, for the observed data and wR2 = 5.62% for all data. The goodness-of-fit was 1.070. The largest peak in the final difference electron density synthesis was 0.331 $e^-/Å^3$ and the largest hole was -0.147 $e^-/Å^3$ with an RMS deviation of 0.038 $e^-/Å^3$. On the basis of the final model, the calculated density was 1.559 g/cm^3 and F(000), 504 e^-.

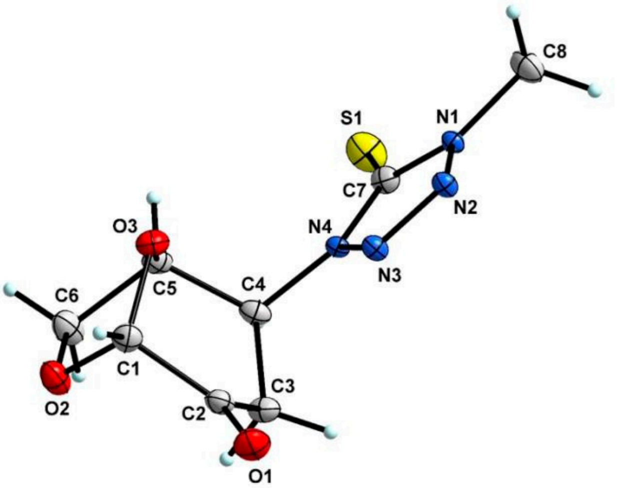

Table S1. Sample and crystal data for compound 6.

**Identification code**	ZJW41	
**Chemical formula**	$C_8H_{10}N_4O_3S$	
**Formula weight**	242.26 g/mol	
**Temperature**	150(2) K	
**Wavelength**	0.71073 Å	
**Crystal system**	monoclinic	
**Space group**	C 1 2 1	
**Unit cell dimensions**	a = 17.019(2) Å	α = 90°
	b = 6.7825(9) Å	β = 114.874(2)°
	c = 9.8582(13) Å	γ = 90°
**Volume**	1032.4(2) $Å^3$	
**Z**	4	
**Density (calculated)**	1.559 g/cm^3	
**Absorption coefficient**	0.312 mm^{-1}	
**F(000)**	504	

Table S2. Data collection and structure refinement for compound 6.

**Theta range for data collection**	2.28 to 30.52°	
**Index ranges**	-24<=h<=24, -9<=k<=9, -14<=l<=14	
**Reflections collected**	16113	
**Independent reflections**	3138 [R(int) = 0.0153]	
**Max. and min. transmission**	0.7461 and 0.7156	
**Refinement method**	Full-matrix least-squares on F^2	
**Refinement program**	SHELXL-2018/3 (Sheldrick, 2018)	
**Function minimized**	$\Sigma w(F_o^2 - F_c^2)^2$	
**Data / restraints / parameters**	3138 / 1 / 185	
**Goodness-of-fit on F^2**	1.070	
**Δ/σ_{max}**	0.001	
**Final R indices**	3100 data; $I>2\sigma(I)$	R1 = 0.0200, wR2 = 0.0560
	all data	R1 = 0.0202, wR2 = 0.0562
**Weighting scheme**	$w=1/[\sigma^2(F_o^2)+(0.0415P)^2+0.1273P]$ where $P=(F_o^2+2F_c^2)/3$	
**Absolute structure parameter**	-0.012(14)	
**Largest diff. peak and hole**	0.331 and -0.147 $eÅ^{-3}$	
**Flack parameter**	-0.012(14)	
**R.M.S. deviation from mean**	0.038 $eÅ^{-3}$	

Table S3. Atomic coordinates and equivalent isotropic atomic displacement parameters (Å^2) for compound 6.

U(eq) is defined as one third of the trace of the orthogonalized U_{ij} tensor.

	x/a	y/b	z/c	U(eq)
S1	0.68001(2)	0.42585(5)	0.32831(3)	0.02417(8)
O1	0.64264(7)	0.71492(15)	0.94822(11)	0.02264(18)
O2	0.61625(7)	0.23095(14)	0.88990(10)	0.02153(18)
O3	0.53899(5)	0.39358(13)	0.67178(9)	0.01686(16)
N1	0.60068(6)	0.76649(15)	0.35338(11)	0.01607(18)
N2	0.57937(7)	0.86089(16)	0.45478(12)	0.01870(19)
N3	0.60485(7)	0.75387(16)	0.57229(11)	0.01799(19)
N4	0.64278(6)	0.59035(15)	0.54790(10)	0.01468(17)
C1	0.57514(7)	0.4121(2)	0.82962(12)	0.01688(18)
C2	0.64396(7)	0.57373(18)	0.87342(11)	0.01526(19)
C3	0.71400(7)	0.53745(18)	0.81969(13)	0.0167(2)
C4	0.67790(6)	0.43853(19)	0.66446(11)	0.01511(18)
C5	0.60653(7)	0.29045(17)	0.64878(12)	0.01593(19)
C6	0.63596(9)	0.13890(18)	0.77497(14)	0.0214(2)
C7	0.64139(7)	0.59232(17)	0.40885(12)	0.01463(19)
C8	0.57942(9)	0.8556(2)	0.20812(14)	0.0223(2)

Table S4. Bond lengths (Å) for compound 6.

S1-C7	1.6662(11)	O1-C2	1.2146(15)
O2-C1	1.4152(16)	O2-C6	1.4510(16)
O3-C1	1.4178(13)	O3-C5	1.4426(14)
N1-N2	1.3585(14)	N1-C7	1.3623(15)
N1-C8	1.4528(16)	N2-N3	1.2784(14)
N3-N4	1.3547(14)	N4-C7	1.3611(14)
N4-C4	1.4698(14)	C1-C2	1.5278(17)
C1-H1	0.974(18)	C2-C3	1.5143(16)
C3-C4	1.5422(16)	C3-H3A	0.95(2)
C3-H3B	0.96(2)	C4-C5	1.5330(17)
C4-H4	0.947(18)	C5-C6	1.5267(17)
C5-H5	0.972(19)	C6-H6A	0.96(2)
C6-H6B	1.00(2)	C8-H8A	0.99(3)
C8-H8B	0.99(3)	C8-H8C	0.94(2)

Table S5. Bond angles (°) for compound 6.

C1-O2-C6	106.35(9)	C1-O3-C5	102.05(8)
N2-N1-C7	111.19(9)	N2-N1-C8	119.76(10)
C7-N1-C8	129.05(10)	N3-N2-N1	107.78(10)
N2-N3-N4	108.02(9)	N3-N4-C7	111.28(9)
N3-N4-C4	120.15(9)	C7-N4-C4	128.56(9)
O2-C1-O3	107.07(10)	O2-C1-C2	108.63(9)
O3-C1-C2	107.52(9)	O2-C1-H1	109.4(14)
O3-C1-H1	111.0(11)	C2-C1-H1	113.0(14)
O1-C2-C3	123.97(11)	O1-C2-C1	121.97(11)
C3-C2-C1	114.03(10)	C2-C3-C4	111.97(9)
C2-C3-H3A	110.9(12)	C4-C3-H3A	111.7(13)
C2-C3-H3B	105.9(12)	C4-C3-H3B	108.3(14)
H3A-C3-H3B	107.8(18)	N4-C4-C5	109.62(8)
N4-C4-C3	109.40(10)	C5-C4-C3	110.68(9)
N4-C4-H4	107.1(11)	C5-C4-H4	107.2(11)
C3-C4-H4	112.8(11)	O3-C5-C6	102.03(9)
O3-C5-C4	108.45(9)	C6-C5-C4	112.61(9)
O3-C5-H5	109.2(11)	C6-C5-H5	114.3(13)
C4-C5-H5	109.8(12)	O2-C6-C5	104.02(10)
O2-C6-H6A	110.9(12)	C5-C6-H6A	110.5(13)
O2-C6-H6B	112.3(14)	C5-C6-H6B	112.2(14)
H6A-C6-H6B	106.9(19)	N4-C7-N1	101.72(9)
N4-C7-S1	129.05(9)	N1-C7-S1	129.22(9)
N1-C8-H8A	107.1(15)	N1-C8-H8B	112.2(17)
H8A-C8-H8B	106.(2)	N1-C8-H8C	104.5(15)
H8A-C8-H8C	122.(2)	H8B-C8-H8C	105.(2)

Table S6. Anisotropic atomic displacement parameters (Å^2) for compound 6.

The anisotropic atomic displacement factor exponent takes the form: $-2\pi^2[h^2 a^{*2} U_{11} + ... + 2 h k a^* b^* U_{12}]$

	**U11**	**U22**	**U33**	**U23**	**U13**	**U12**
S1	0.03065(15)	0.02569(15)	0.02270(14)	-0.00159(12)	0.01759(11)	0.00760(13)
O1	0.0296(5)	0.0211(4)	0.0177(4)	-0.0064(3)	0.0104(3)	-0.0014(3)
O2	0.0316(5)	0.0177(4)	0.0169(4)	0.0019(3)	0.0117(3)	0.0021(3)
O3	0.0143(3)	0.0191(4)	0.0150(3)	-0.0022(3)	0.0040(3)	-0.0002(3)
N1	0.0173(4)	0.0180(4)	0.0144(4)	0.0002(3)	0.0082(3)	0.0010(3)
N2	0.0235(5)	0.0178(4)	0.0172(4)	0.0001(3)	0.0109(4)	0.0035(4)
N3	0.0224(4)	0.0167(4)	0.0167(4)	-0.0005(3)	0.0099(4)	0.0048(4)
N4	0.0158(4)	0.0163(4)	0.0132(4)	-0.0010(3)	0.0072(3)	0.0019(3)
C1	0.0183(4)	0.0169(4)	0.0162(4)	-0.0013(4)	0.0081(3)	-0.0004(4)
C2	0.0167(4)	0.0166(5)	0.0103(4)	-0.0002(4)	0.0036(4)	0.0006(4)
C3	0.0141(4)	0.0210(5)	0.0130(4)	-0.0018(4)	0.0038(4)	-0.0005(4)
C4	0.0149(4)	0.0171(4)	0.0126(4)	0.0003(4)	0.0050(3)	0.0036(4)
C5	0.0188(4)	0.0143(4)	0.0130(4)	-0.0013(4)	0.0050(4)	0.0013(4)
C6	0.0313(6)	0.0148(5)	0.0183(5)	0.0003(4)	0.0105(4)	0.0039(4)
C7	0.0136(4)	0.0174(5)	0.0140(4)	-0.0016(4)	0.0070(3)	-0.0004(4)
C8	0.0242(6)	0.0275(6)	0.0168(5)	0.0054(4)	0.0103(4)	0.0020(5)

Table S7. Hydrogen atomic coordinates and isotropic atomic displacement parameters (Å^2) for compound 6.

	**x/a**	**y/b**	**z/c**	**U(eq)**
H1	0.5303(12)	0.438(4)	0.864(2)	0.024(4)
H4	0.7208(11)	0.369(3)	0.6463(19)	0.017(4)
H3A	0.7441(13)	0.656(3)	0.821(2)	0.023(5)
H3B	0.7541(14)	0.449(4)	0.890(2)	0.031(5)
H5	0.5826(12)	0.234(3)	0.549(2)	0.022(4)
H6A	0.6043(13)	0.017(3)	0.741(2)	0.027(5)
H6B	0.6986(14)	0.106(4)	0.811(3)	0.031(5)
H8A	0.5155(17)	0.872(4)	0.159(3)	0.048(7)
H8B	0.6043(18)	0.990(4)	0.217(3)	0.050(8)
H8C	0.6091(14)	0.779(4)	0.166(3)	0.038(6)

Levo-tetrazole 6
tried ~35 mg in 0.67 mL CDCl3 with sonication
had to syringe filter to remove udissolved solid

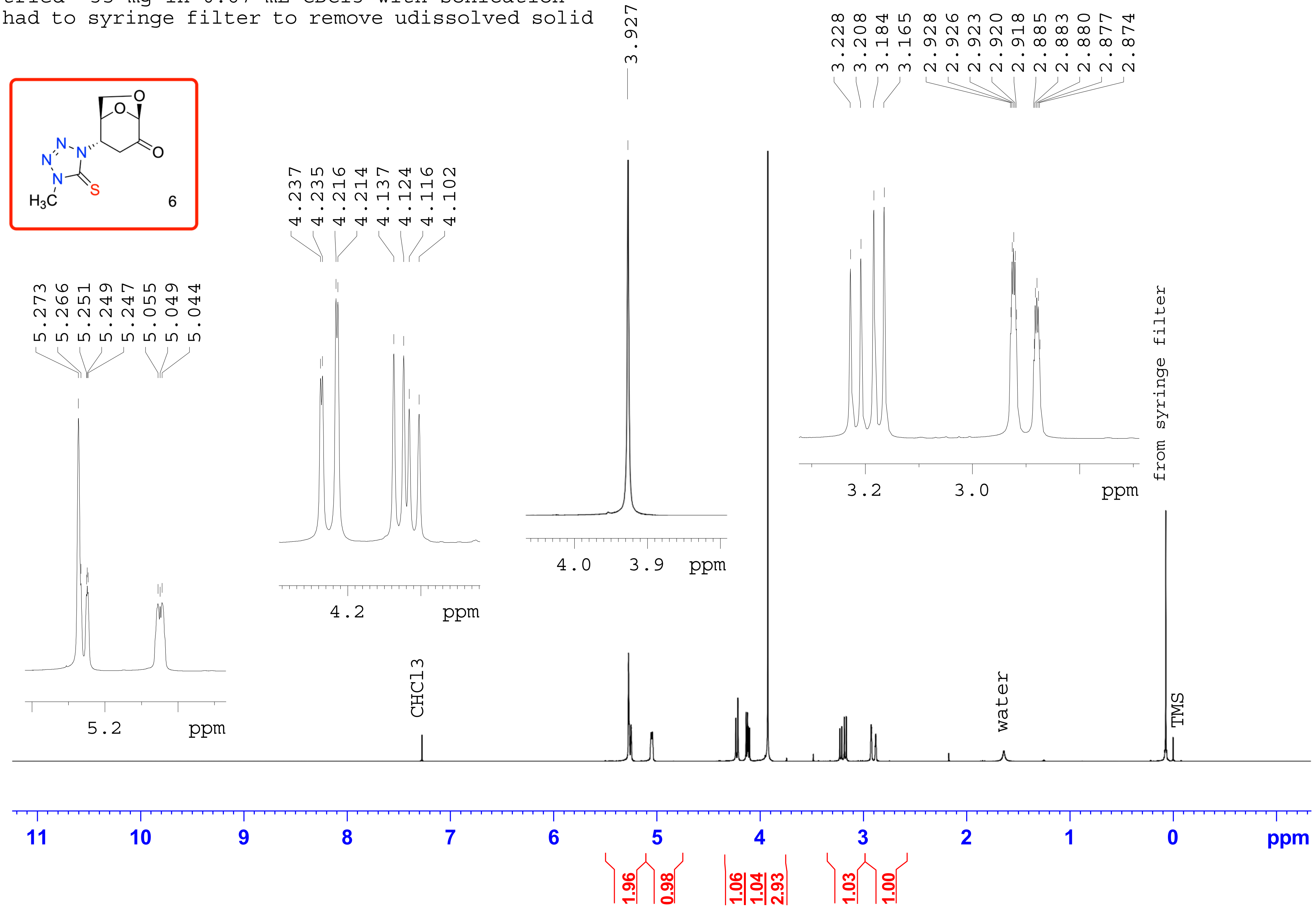

Levo-tetrazole **6**
tried ~35 mg in 0.67 mL CDCl3 with sonication
had to syringe filter to remove udissolved solid

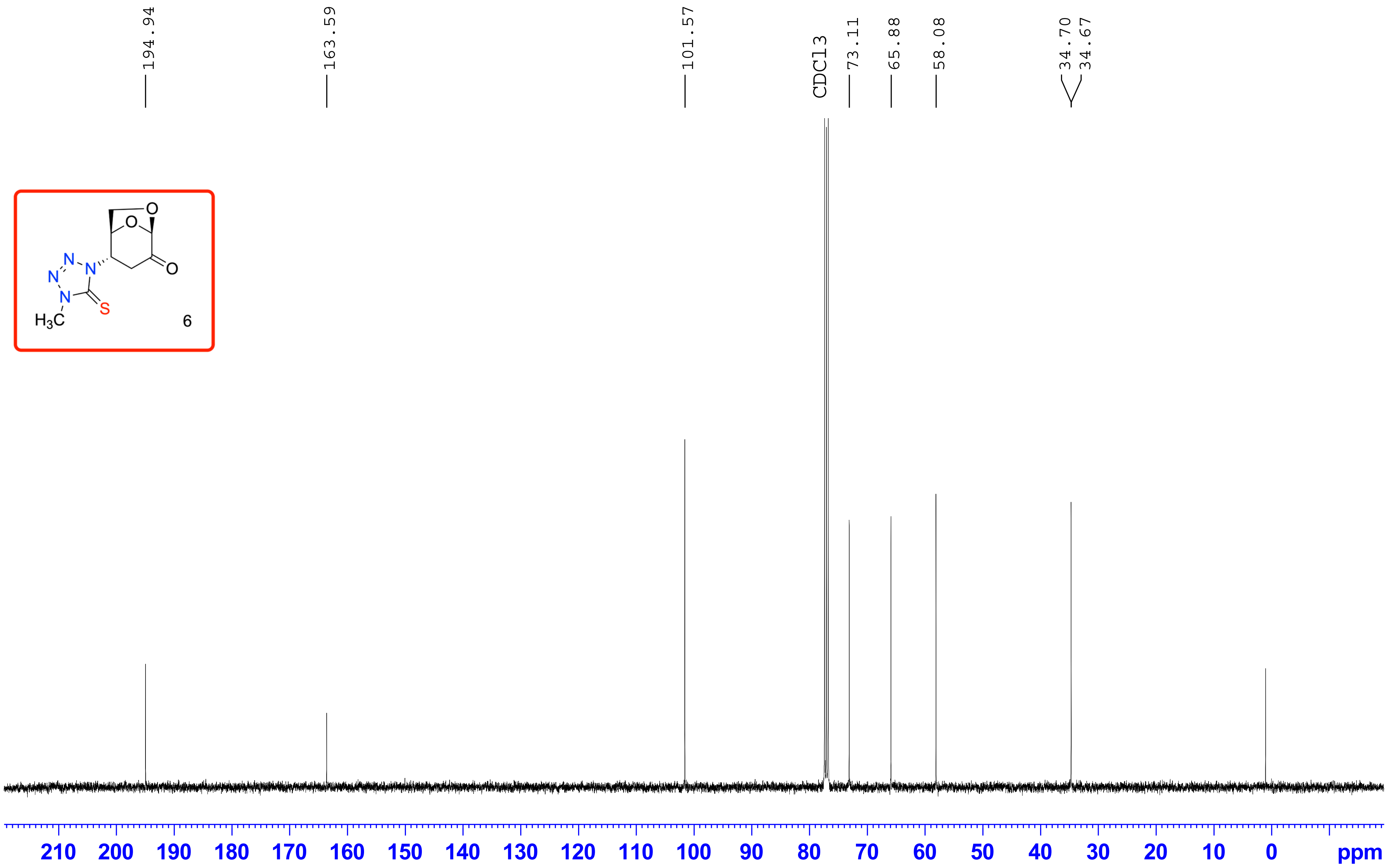

6

Levo-tetrazole 6
tried ~35 mg in 0.67 mL CDCl3 with sonication
had to syringe filter to remove udissolved solid

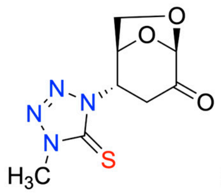

6

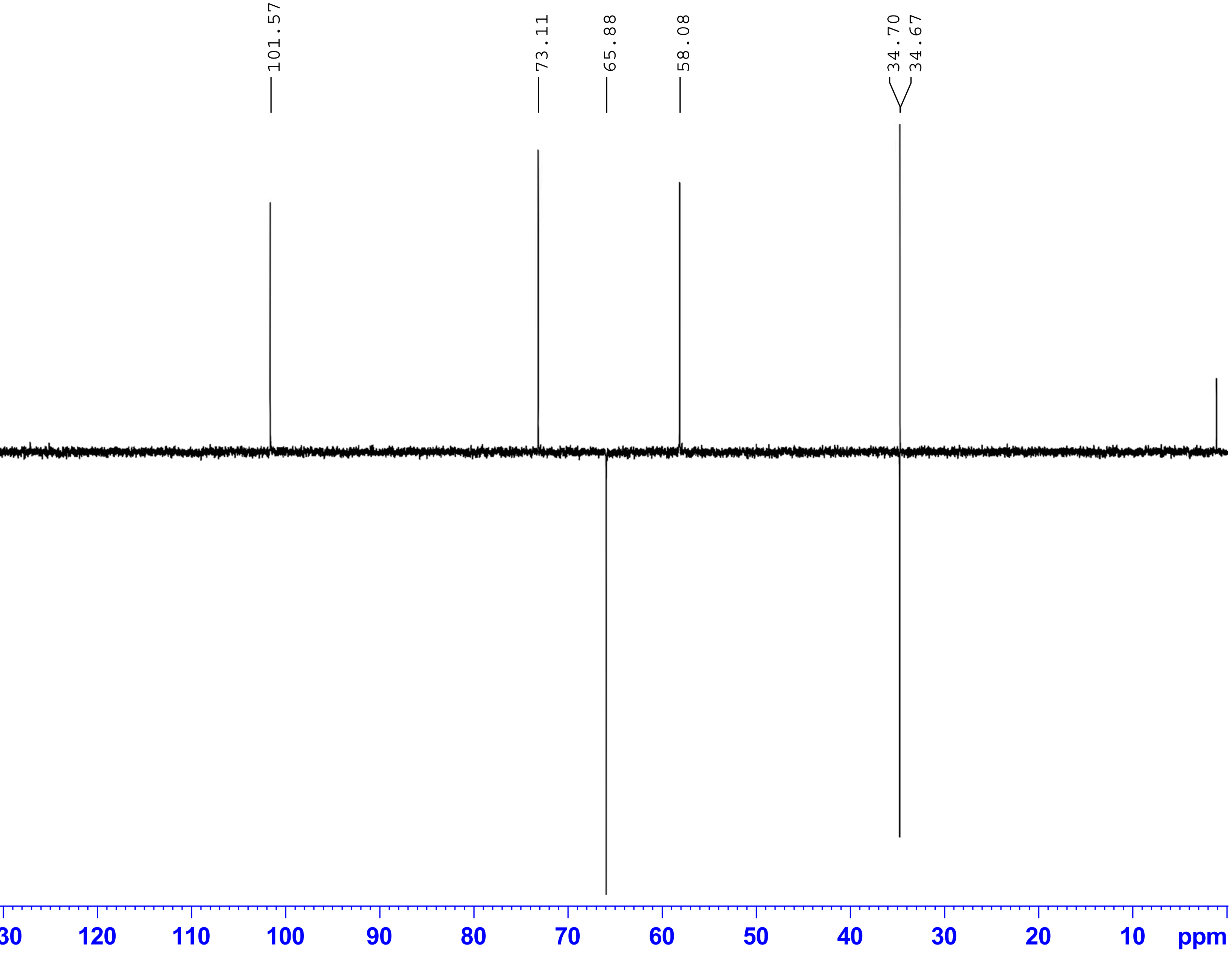

Levo-tetrazole 6
tried ~35 mg in 0.67 mL CDCl3 with sonication
had to syringe filter to remove udissolved solid

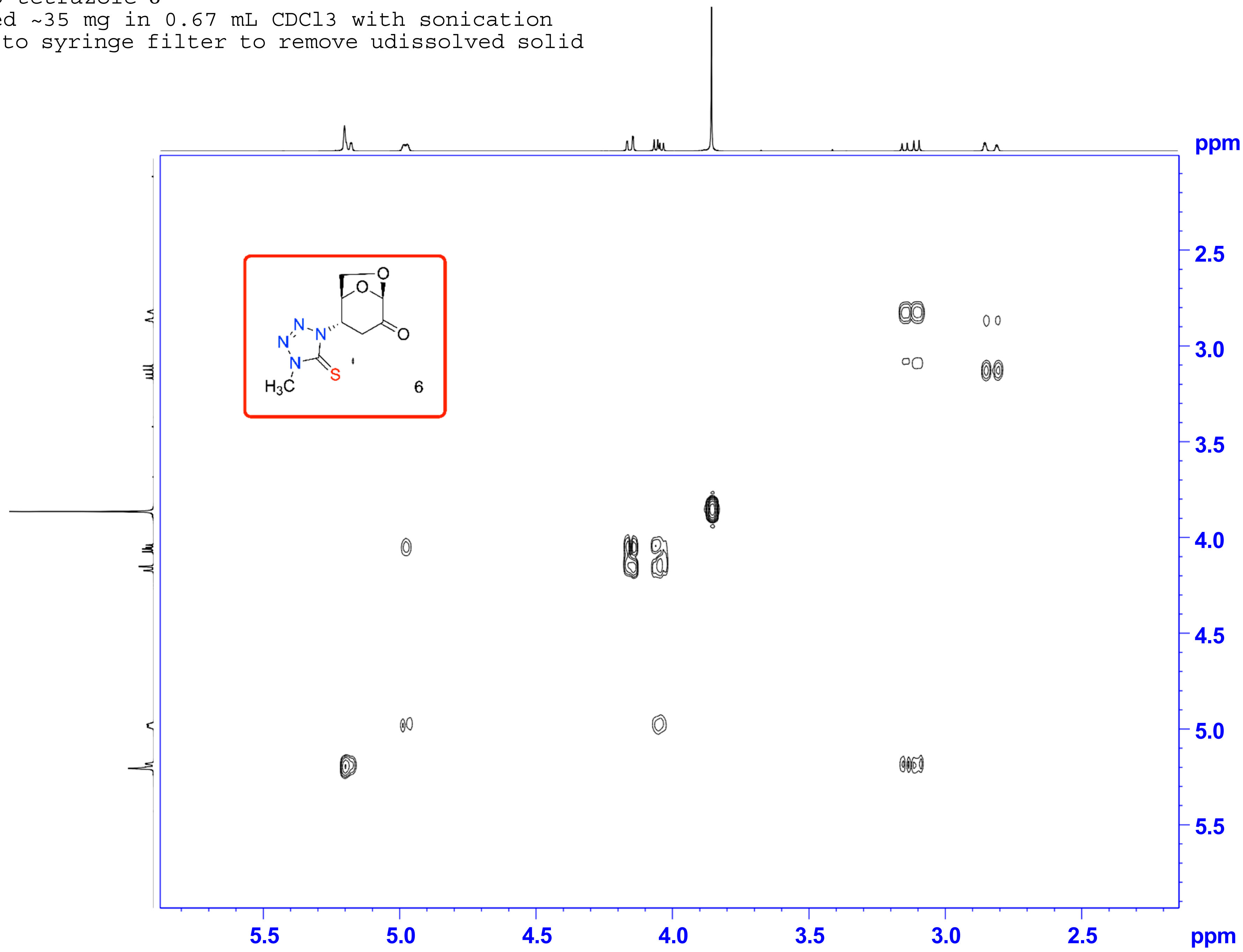

Levo-tetrazole 6
tried ~35 mg in 0.67 mL CDCl3 with sonication had to syringe
filter to remove undissolved solid

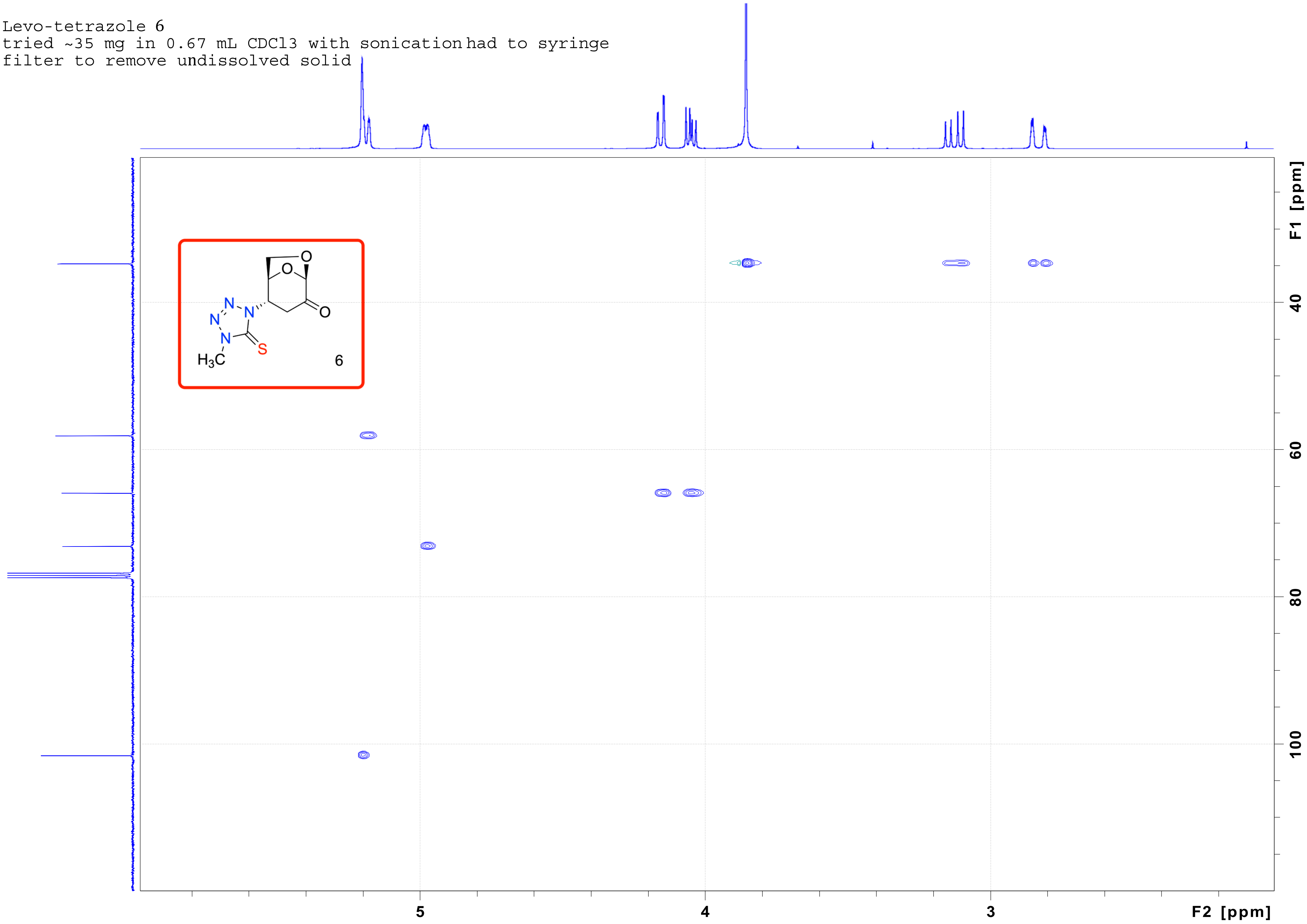

ZJW-125 ~18 mg in ~0.67 mL CDCl3 compound 10

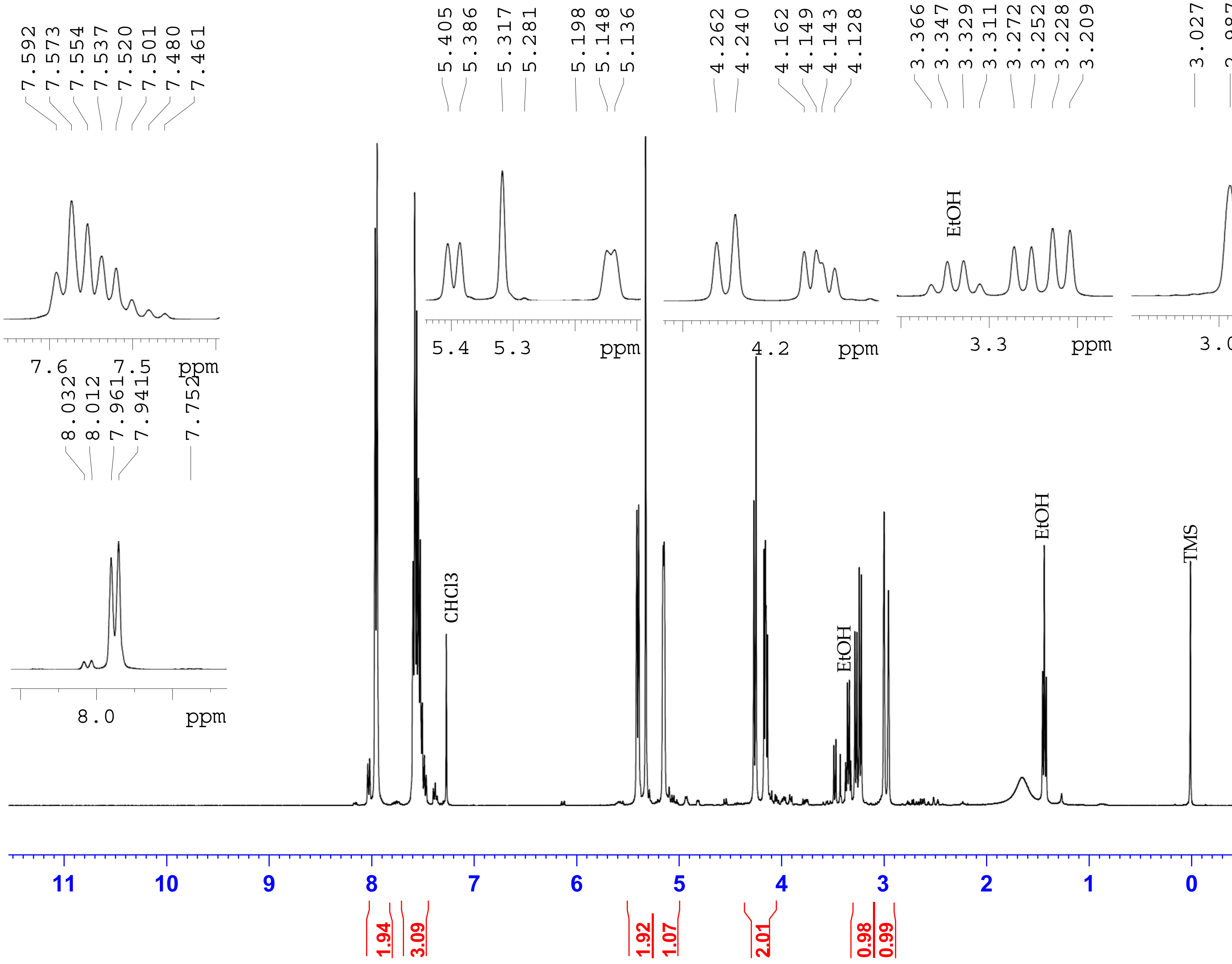

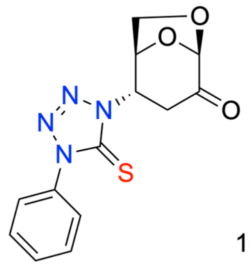

10

ZJW-125 ~18 mg in ~0.67 mL CDCl3 compound 10

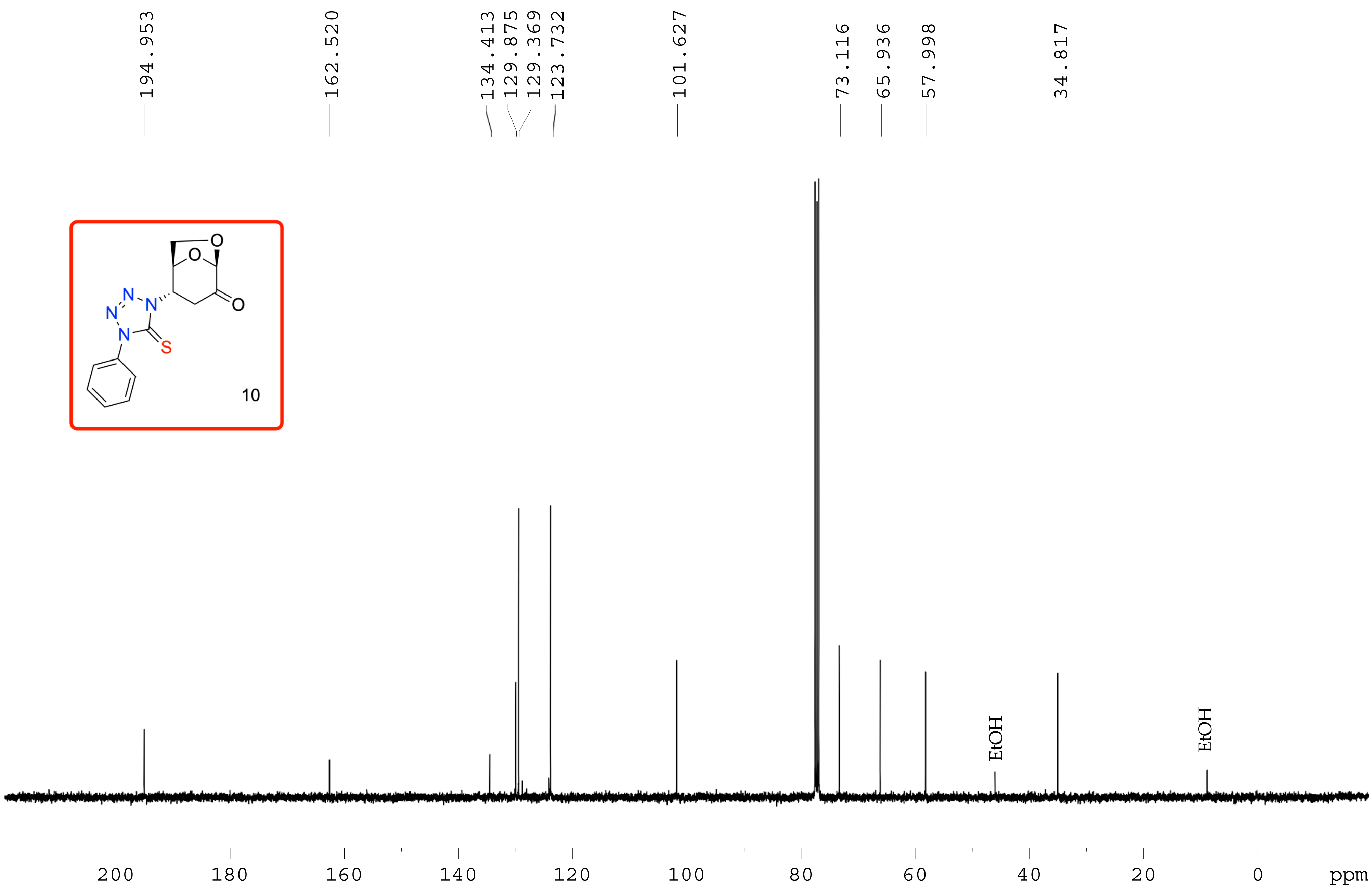

ZJW-125 ~18 mg in ~0.67 mL CDCl3 compound 10

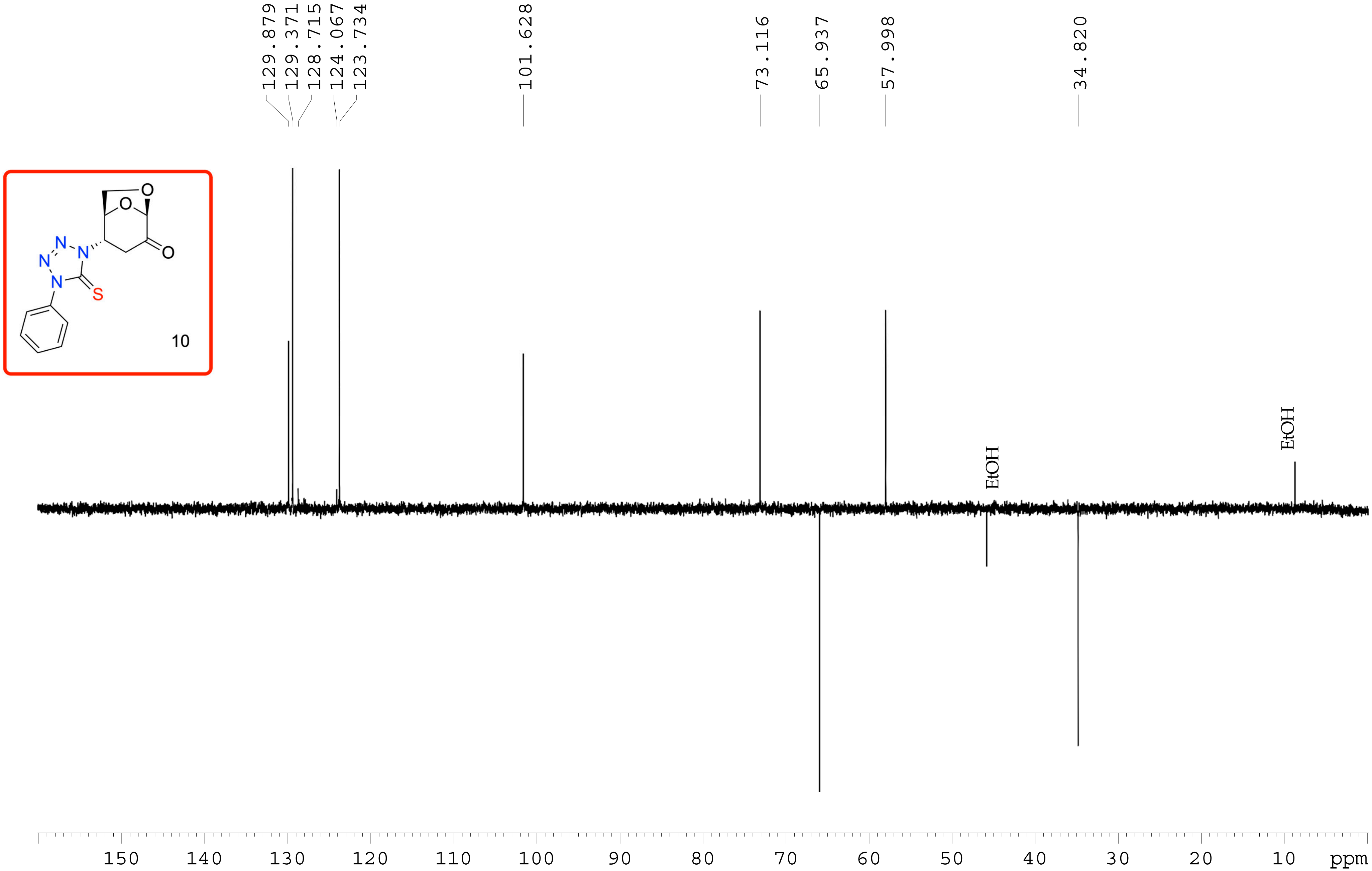

ZJW-125 ~18 mg in ~0.67 mL CDCl3
compound 10

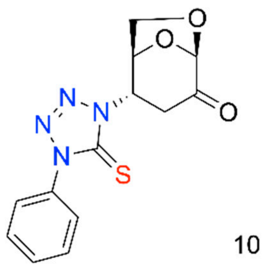

10

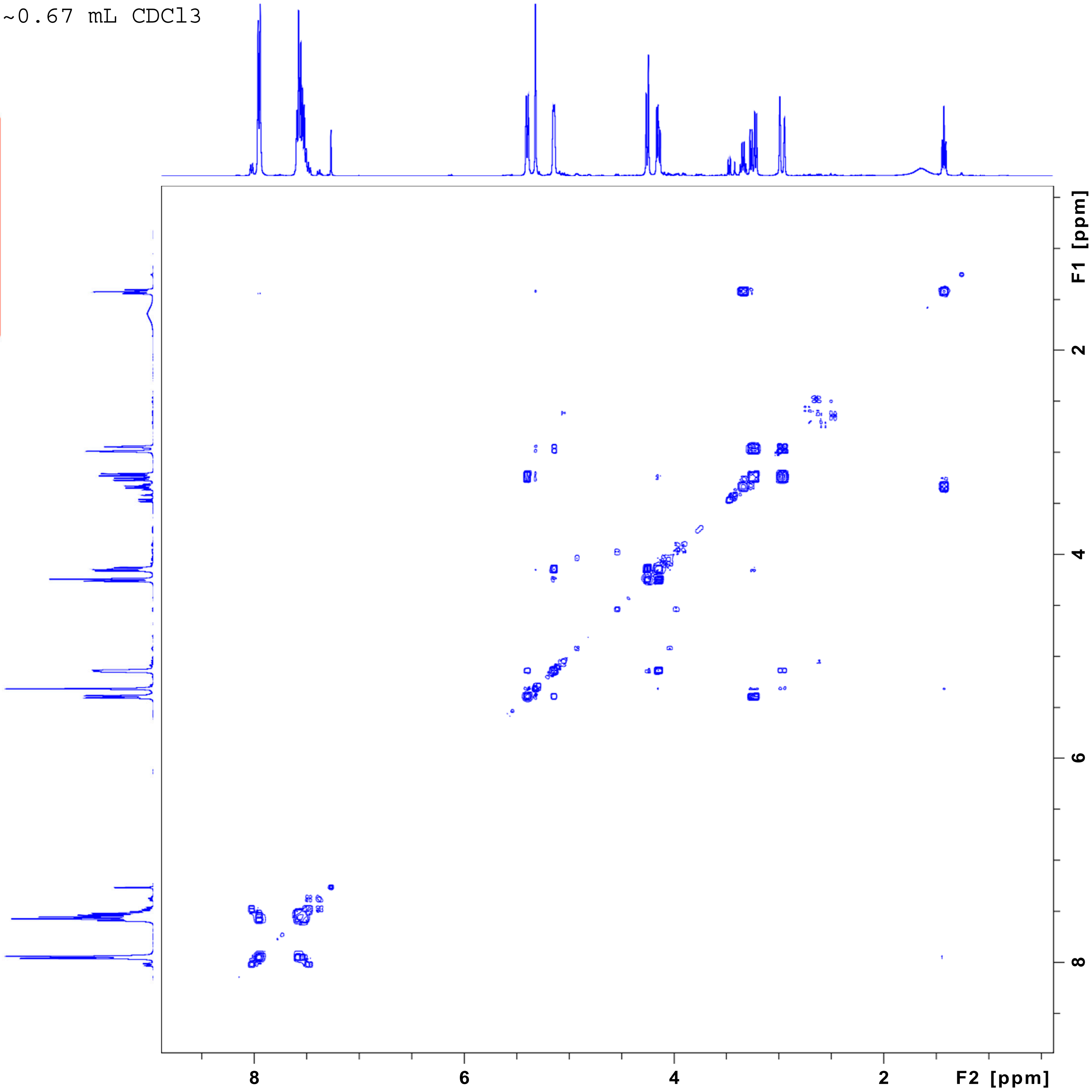

ZJW-125 ~18 mg in ~0.67 mL CDCl3
compound 10

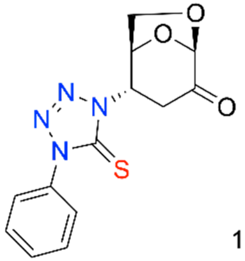

10

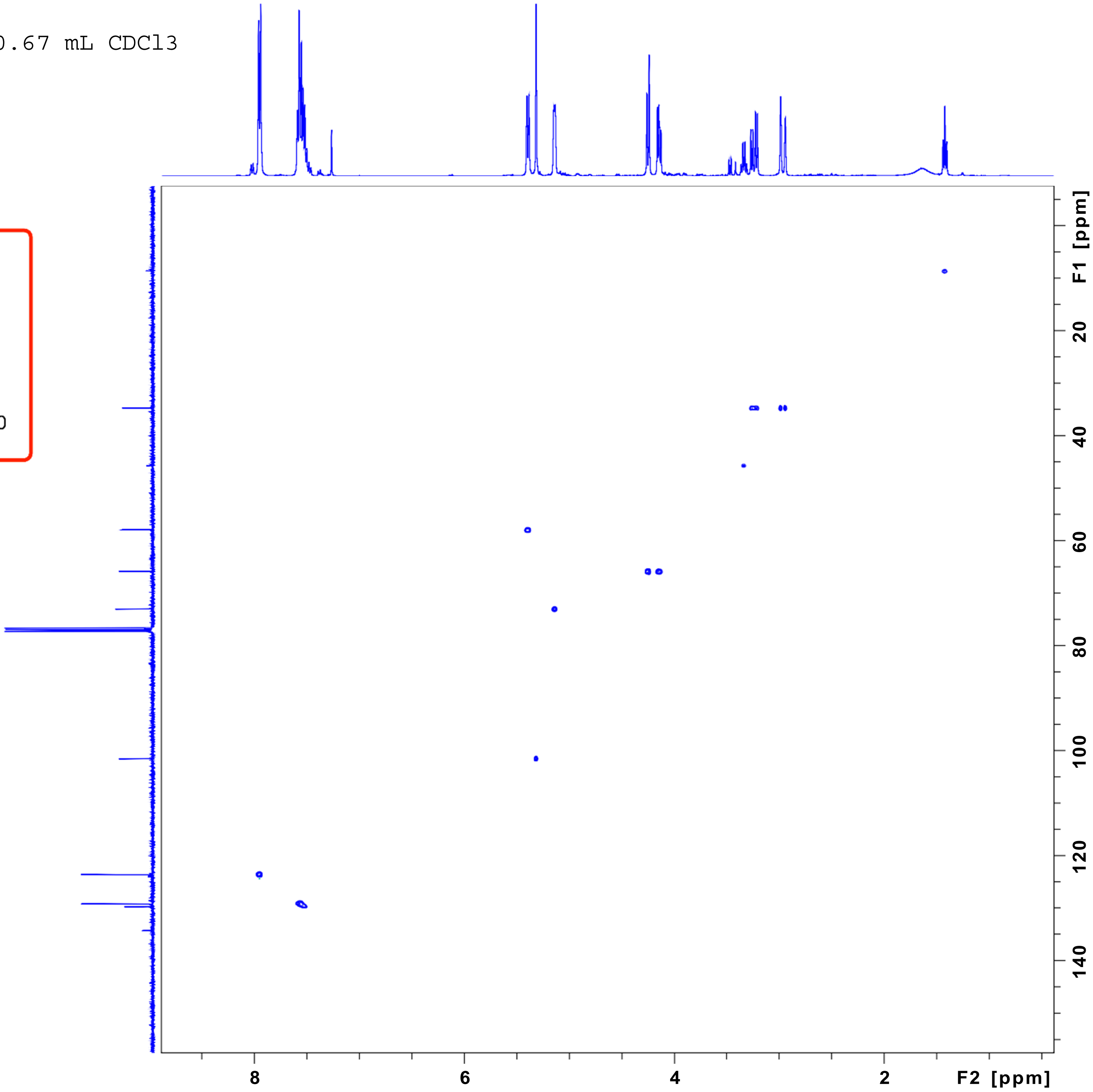

ZJW 127 - II ~19 mg in ~0.67 mL CDCl3 compound 11

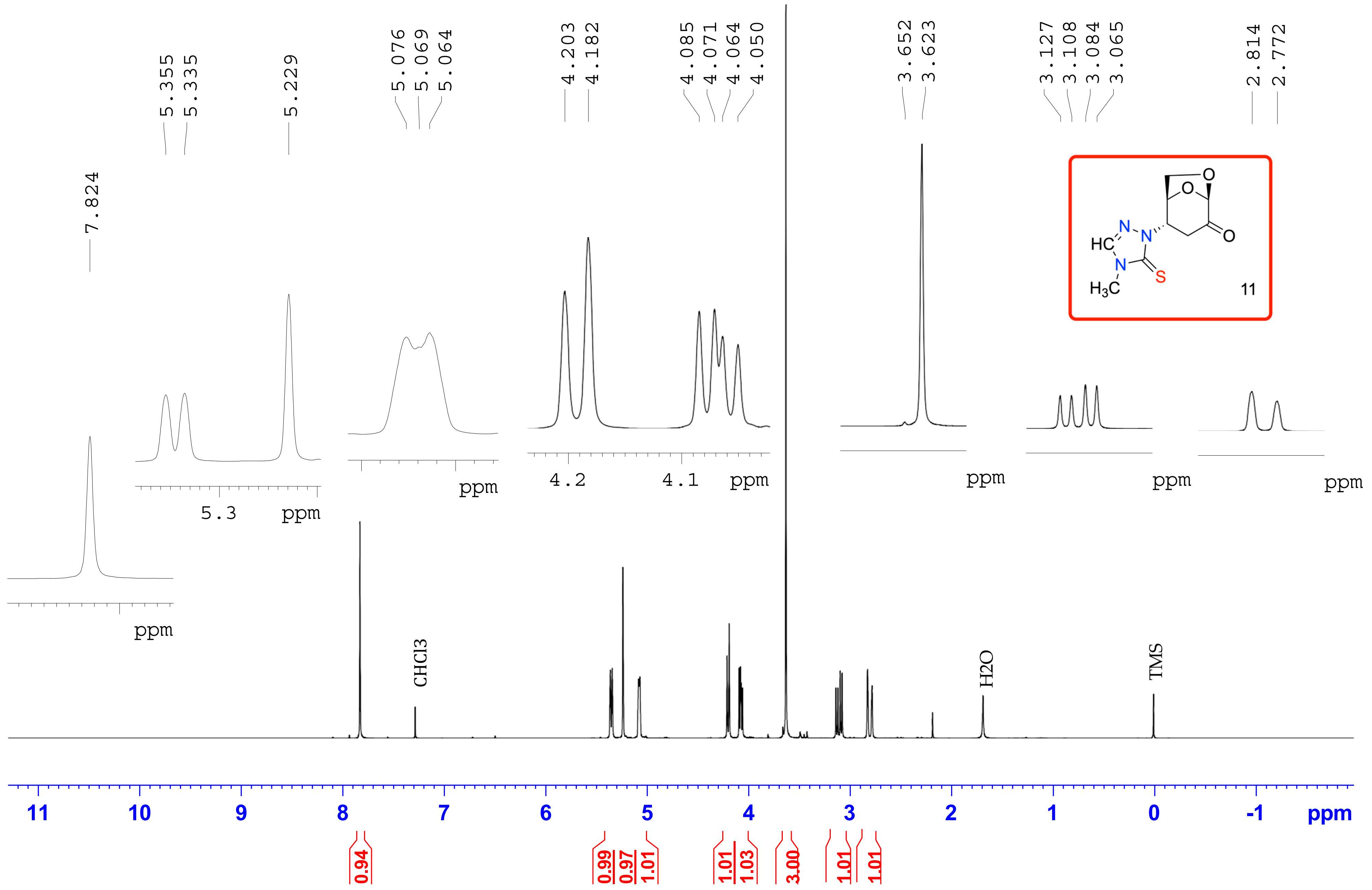

ZJW 127 - II ~19 mg in ~0.67 mL CDCl3 compond 11

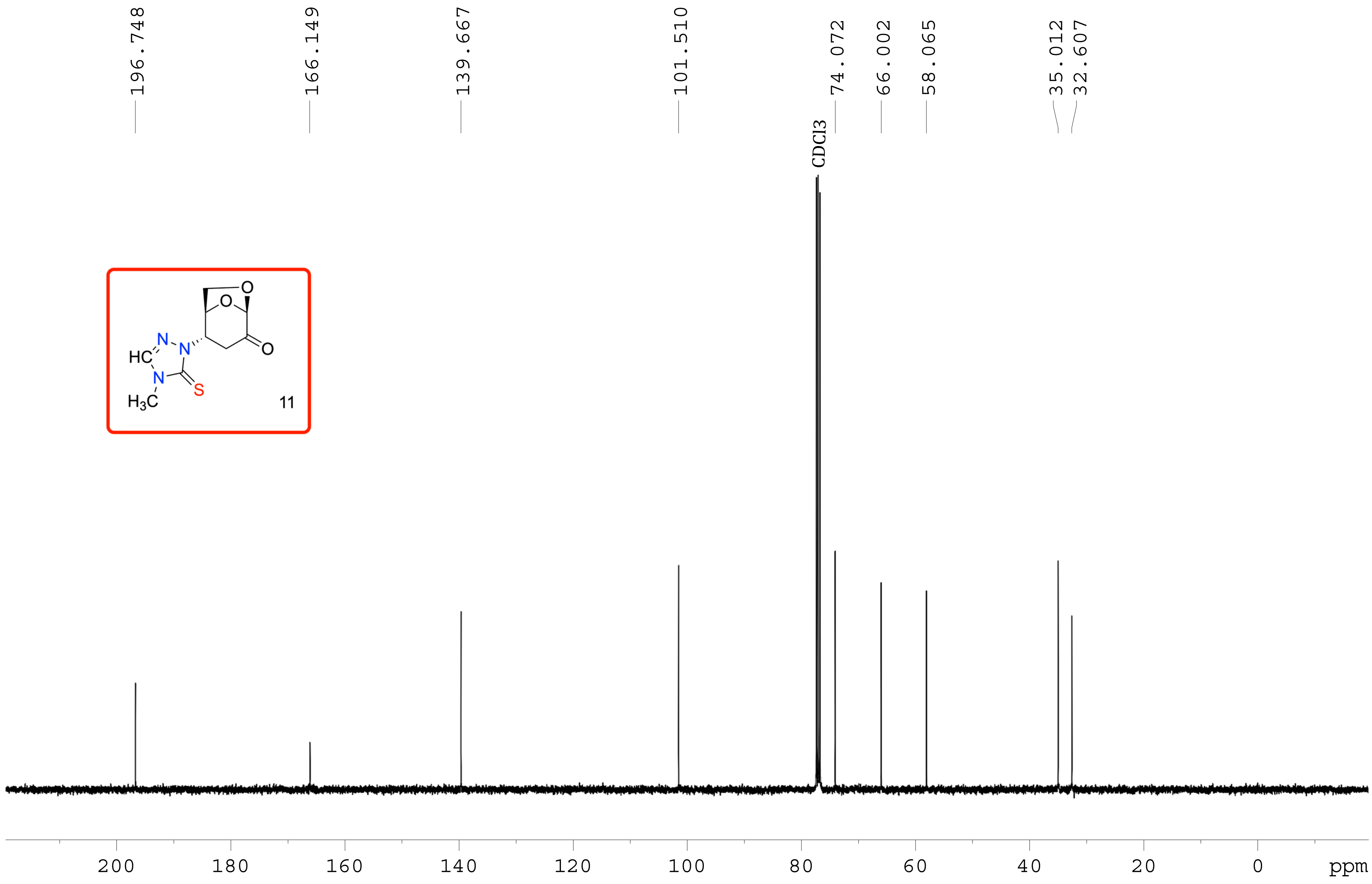

ZJW 127 - II ~19 mg in ~0.67 mL CDCl3 compound 11

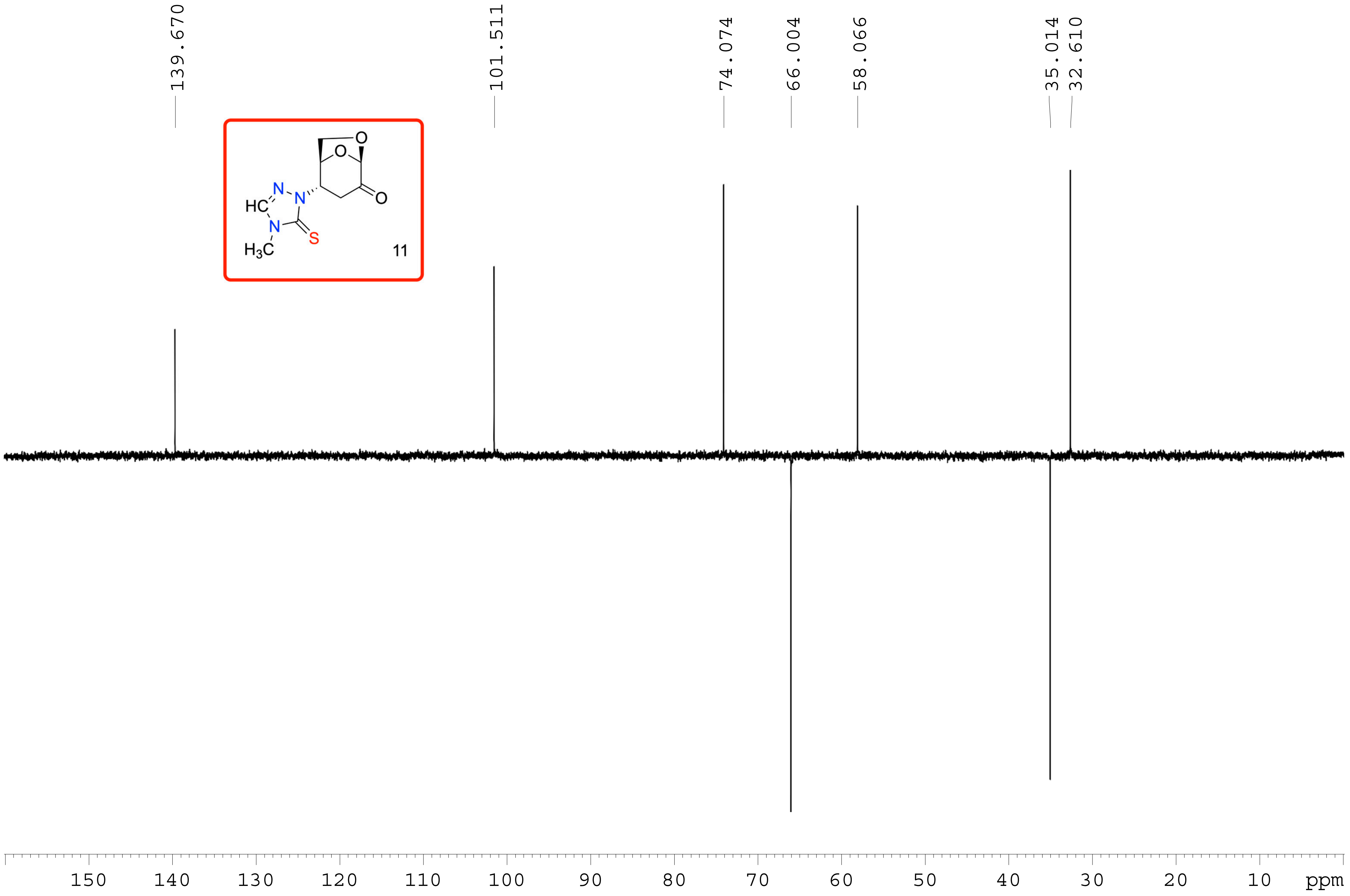

ZJW 127 - II ~19 mg in ~0.67 mL CDCl3 compound 11

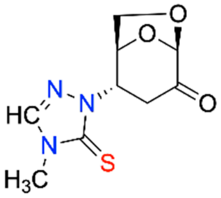

11

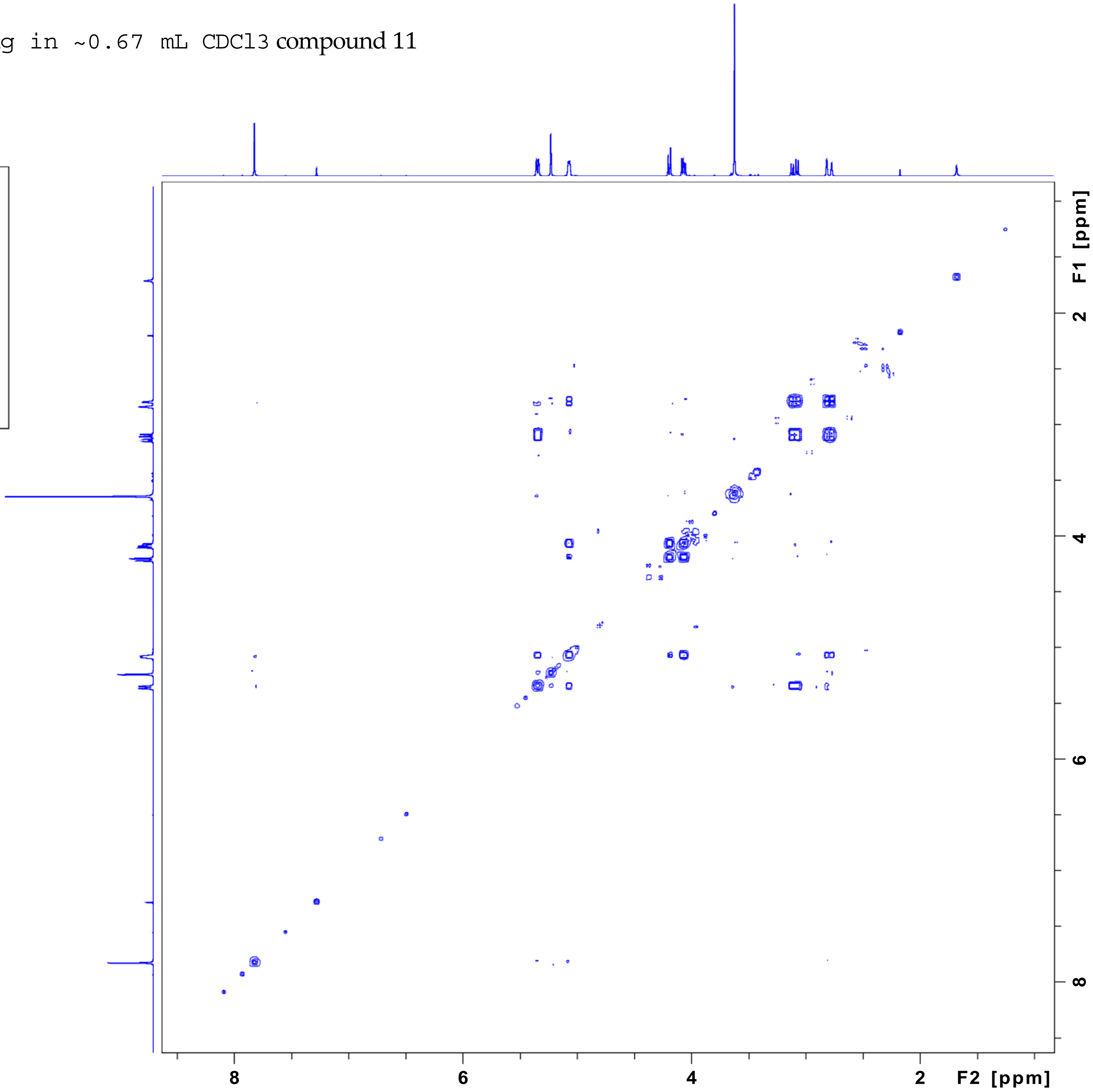

ZJW 127 - II ~19 mg in ~0.67 mL CDCl3 compound 11

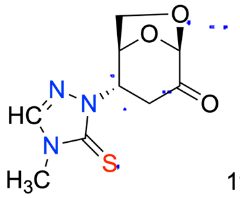

11

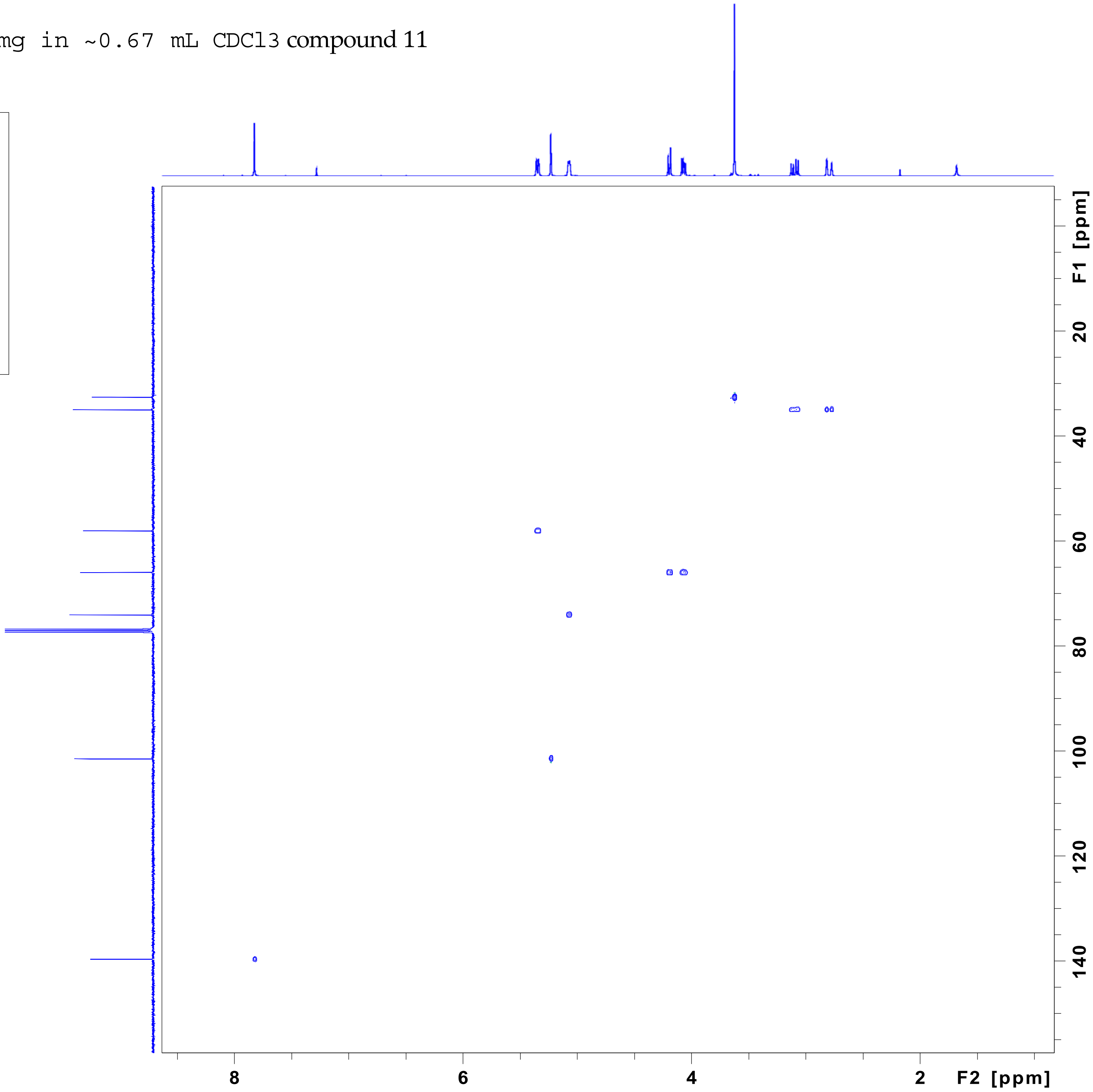

ZJW-130

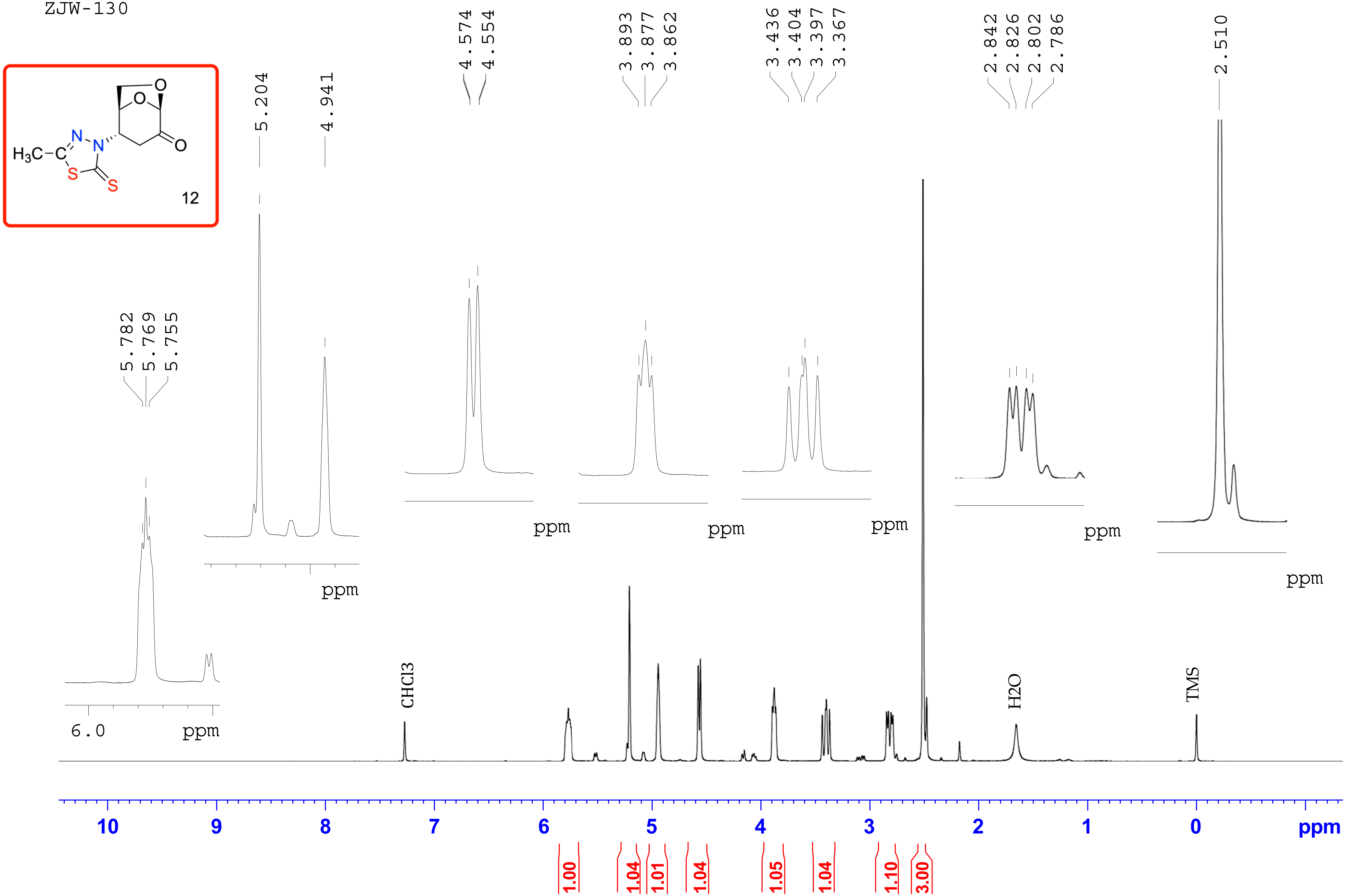

H3C–C
N
N
S
S
O
O
O
12
5.782
5.769
5.755
5.204
4.941
4.574
4.554
3.893
3.877
3.862
3.436
3.404
3.397
3.367
2.842
2.826
2.802
2.786
2.510
6.0
ppm
CHCl3
H2O
TMS
10
9
8
7
6
5
4
3
2
1
0
1.00
1.04
1.01
1.04
1.05
1.04
1.10
3.00

ZJW_130_9Nov2025 12 1 /opt/nmrdata/user/Mencer

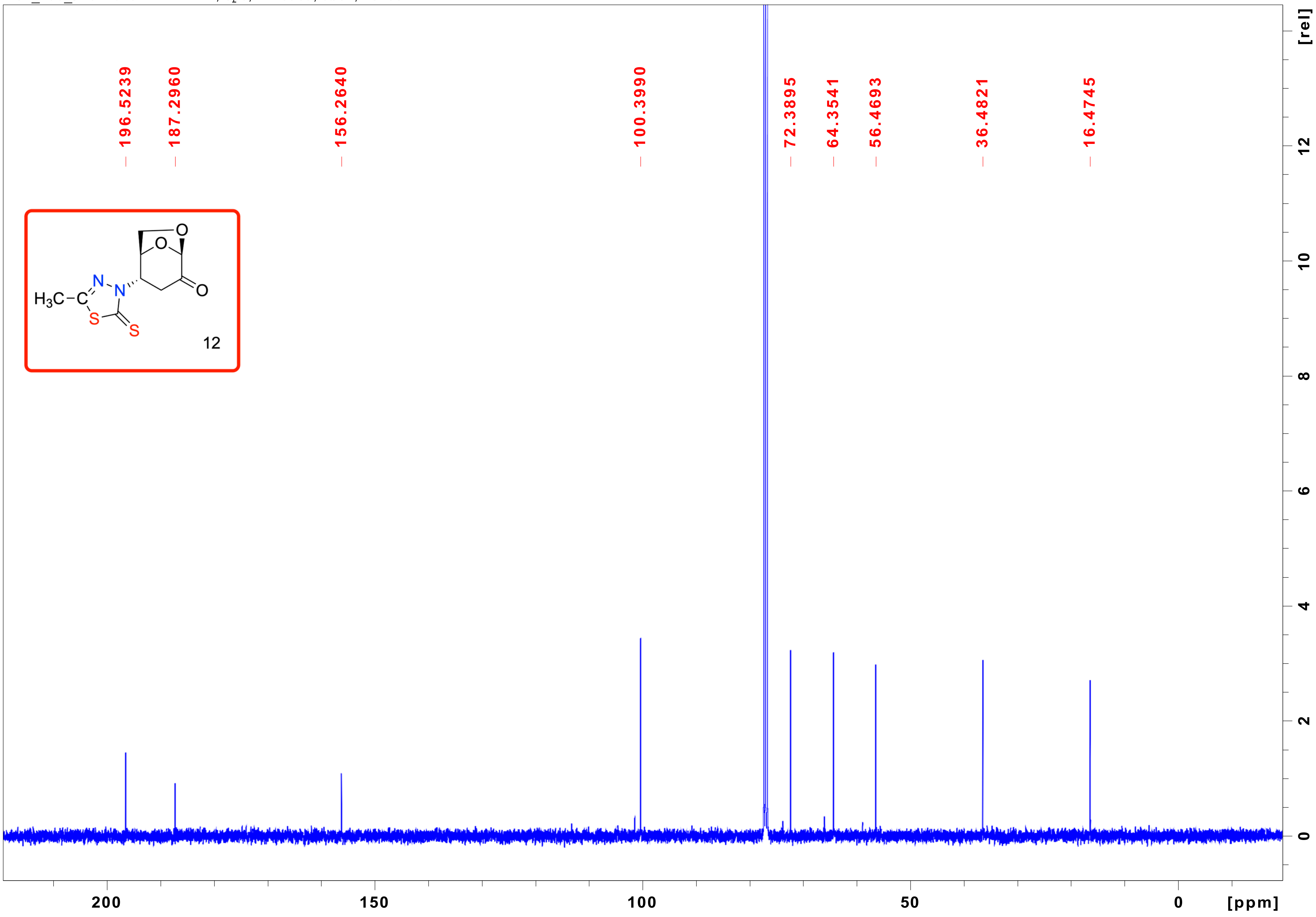

ZJW_130_9Nov2025 14 1 /opt/nmrdata/user/Mencer

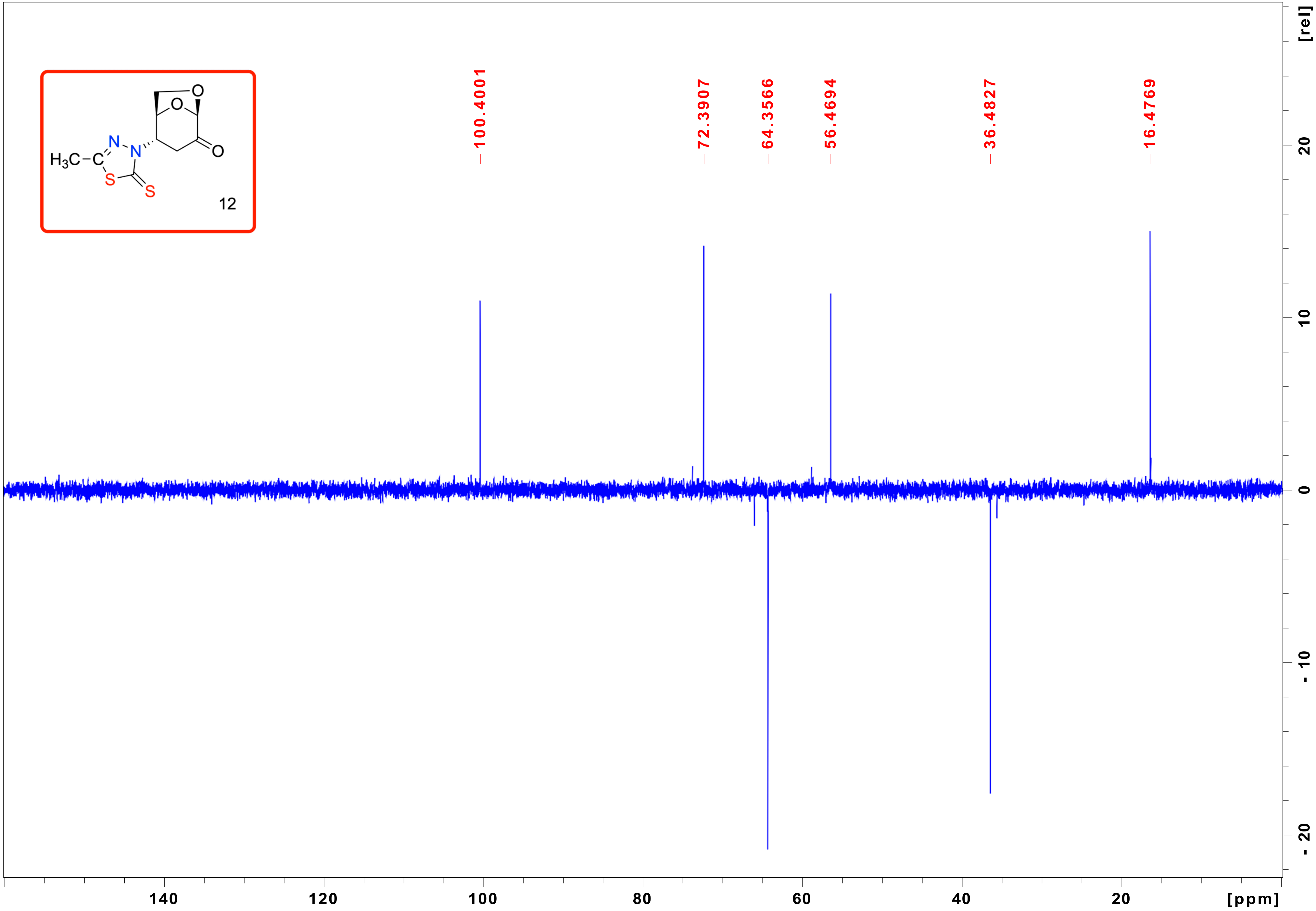

ZJW_130_9Nov2025 11 1 /opt/nmrdata/user/Mencer

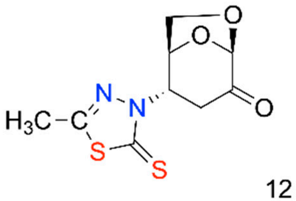

12

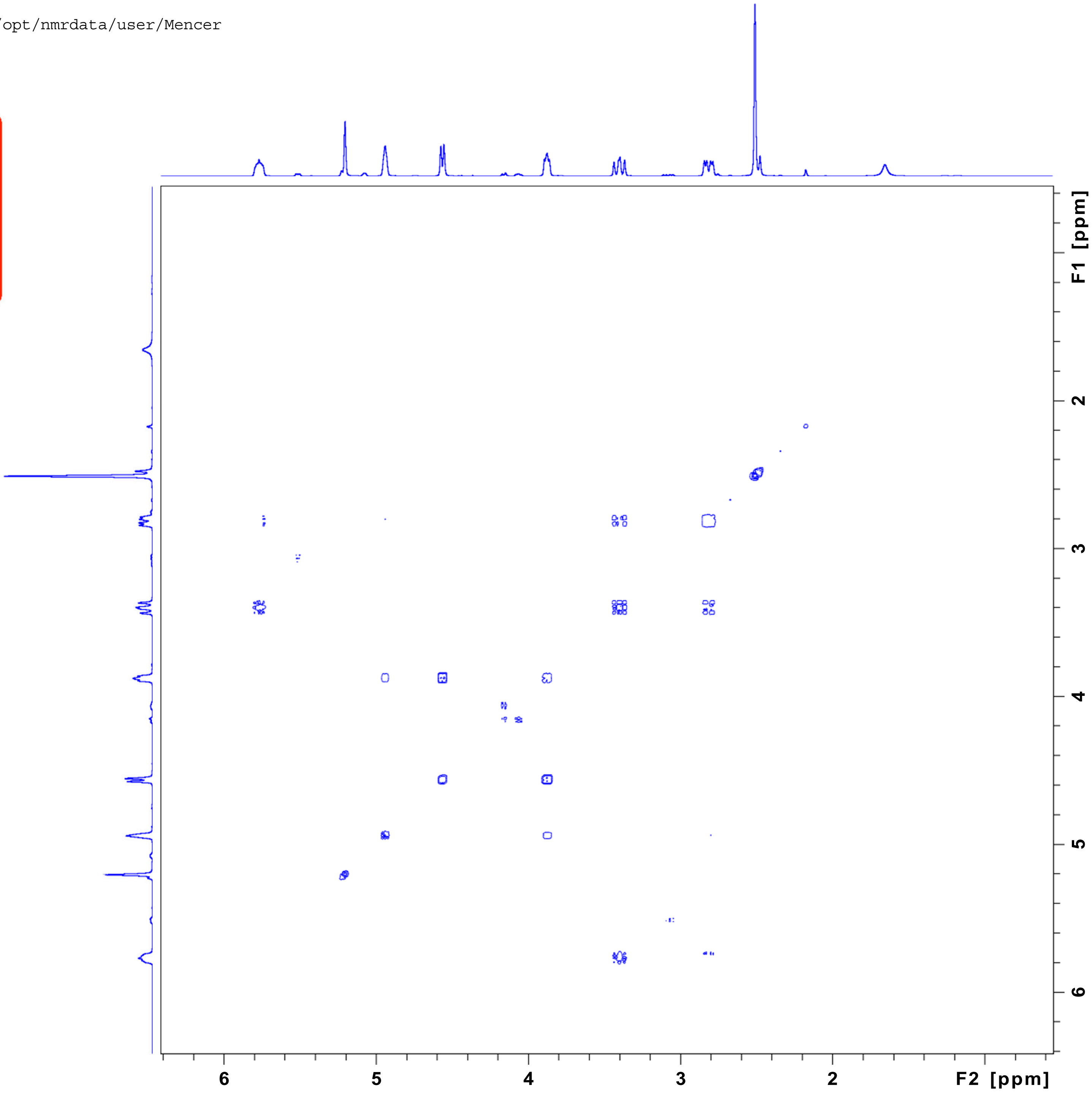

ZJW_130_9Nov2025 13 1 /opt/nmrdata/user/Mencer

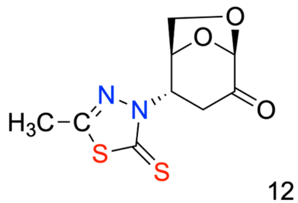

12

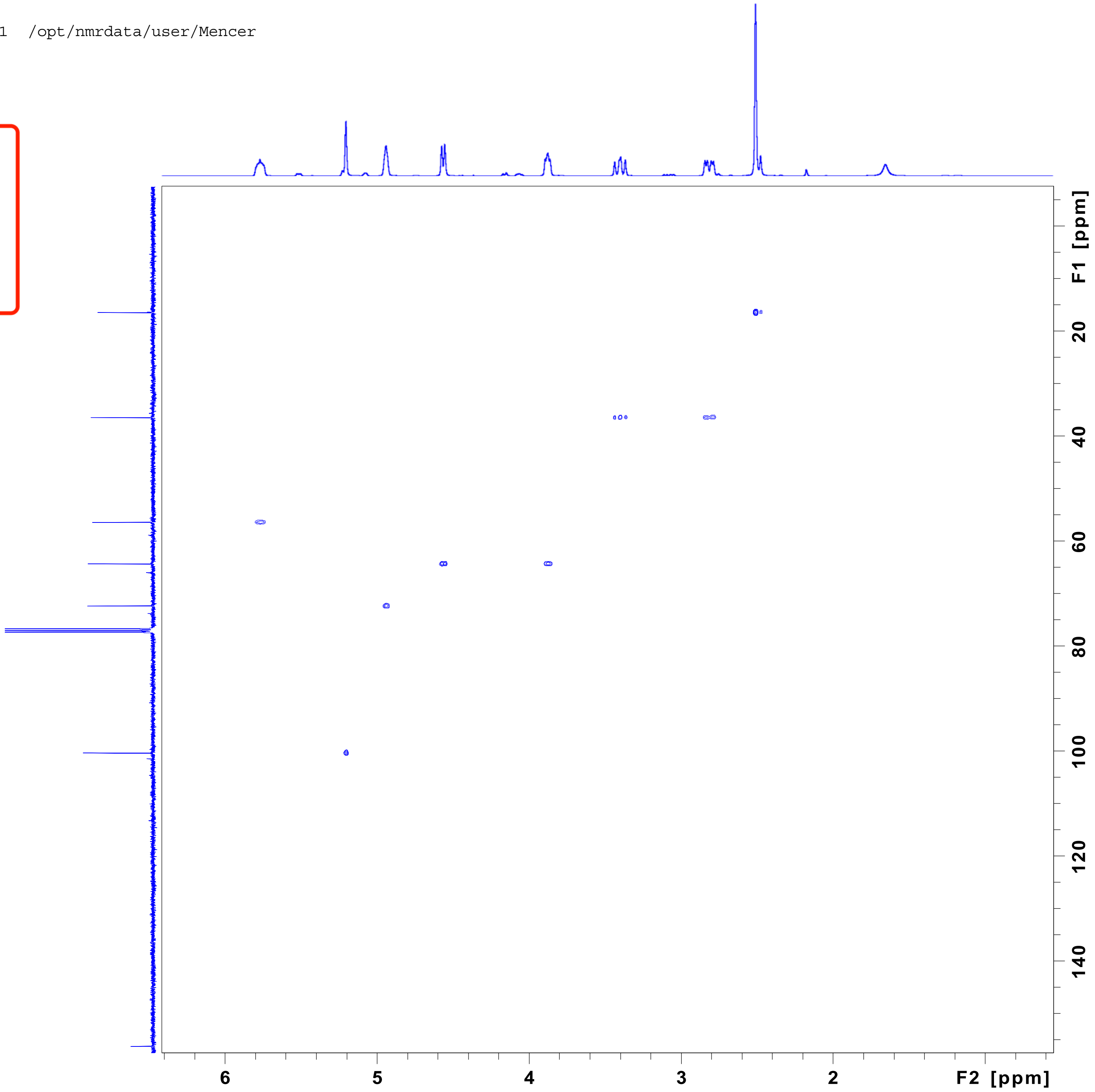

Supplement: Supplementary file 1 [file molecules-31-00164-s001.zip › molecules-4074507-supplementary.pdf]
